# Skewed epithelial cell differentiation and premature aging of the thymus in the absence of vitamin D signaling

**DOI:** 10.1126/sciadv.adm9582

**Published:** 2024-09-25

**Authors:** Patricio Artusa, Loan Nguyen Yamamoto, Camille Barbier, Stefanie F. Valbon, Yashar Aghazadeh Habashi, Haig Djambazian, Aiten Ismailova, Marie-Ève Lebel, Reyhaneh Salehi-Tabar, Fatemeh Sarmadi, Jiannis Ragoussis, David Goltzman, Heather J. Melichar, John H. White

**Affiliations:** ^1^ Department of Physiology, McGill University, Montreal QC, Canada.; ^2^ Department of Medicine, McGill University, Montreal QC, Canada.; ^3^ Calcium Research Laboratory, McGill University Health Centre, Montreal QC, Canada.; ^4^ Department of Microbiology, Immunology and Infectious Disease, Université de Montréal, Montreal, QC, Canada.; ^5^ Rosalind and Morris Goodman Cancer Institute, McGill University, Montreal, QC, Canada.; ^6^ McGill University Genome Centre, McGill University, Montreal, QC, Canada.; ^7^ Maisonneuve-Rosemont Hospital Research Center, McGill University, Montreal, QC, Canada.; ^8^ Department of Microbiology and Immunology, McGill University, Montreal, QC, Canada.

## Abstract

Central tolerance of thymocytes to self-antigen depends on the medullary thymic epithelial cell (mTEC) transcription factor autoimmune regulator (Aire), which drives tissue-restricted antigen (TRA) gene expression. Vitamin D signaling regulates Aire and TRA expression in mTECs, providing a basis for links between vitamin D deficiency and autoimmunity. We find that mice lacking Cyp27b1, which cannot produce hormonally active vitamin D, display profoundly reduced thymic cellularity, with a reduced proportion of Aire^+^ mTECs, attenuated TRA expression, and poorly defined cortical-medullary boundaries. Markers of T cell negative selection are diminished, and organ-specific autoantibodies are present in knockout (KO) mice. Single-cell RNA sequencing revealed that loss of Cyp27b1 skews mTEC differentiation toward Ccl21^+^ intertypical TECs and generates a gene expression profile consistent with premature aging. KO thymi display accelerated involution and reduced expression of thymic longevity factors. Thus, loss of thymic vitamin D signaling disrupts normal mTEC differentiation and function and accelerates thymic aging.

## Teaser

Loss of vitamin D signaling disrupts development and induces premature aging of the thymus.

## INTRODUCTION

The thymus is a critical primary lymphoid organ whose microenvironment supports the multistep process of T lymphopoiesis. This gives rise to T cell populations that can recognize and eliminate foreign antigens but are tolerant of self-peptides (*1*). Two-way communication between thymocytes and the thymic stroma, particularly thymic epithelial cells (TECs), is critical for normal lymphopoiesis. This interplay controls developing T cell migration, proliferation, differentiation, and survival. TECs are organized spatially and functionally into cortical (cTEC) and medullary (mTEC) compartments (*2*). Early T cell developmental checkpoints such as lineage commitment and positive selection are orchestrated by cTECs, whereas subsequent steps, including negative selection of self-reactive cells or differentiation into FoxP3^+^ CD25^+^ regulatory T cells (T_regs_), are largely controlled by mTECs. It is important to note that these processes occur optimally early in life as the thymus is the most rapidly aging organ in the body. The aging thymus undergoes a process of regression, which is accompanied by a loss of cellularity and morphological disorganization (*3*). In mice, this process starts within 4 weeks of life, with the degeneration of the TEC compartment playing a major role (*4*).

TEC populations are not uniform, and mTECs undergo a differentiation process to produce multiple specialized compartments (*2*). mTEC subsets differ in major histocompatibility complex (MHC) and costimulatory molecule expression (*5*–*9*), which are required for negative selection and T_reg_ development (*10*, *11*). mTEC^lo^ (CD80/CD86^lo^ MHC class II^lo^) cells include “immature” progenitors, functionally mature cells, and terminally differentiated TECs (*5*, *7*, *12*). Immature mTEC^lo^ differentiate into an mTEC^hi^ population that expresses higher levels of costimulatory molecules and MHC class II (CD80/CD86^hi^ MHC class II^hi^) (*13*, *14*). Promiscuous gene expression by mTEC populations is critical for tolerance to tissue-restricted antigens (TRAs) (*1*, *15*–*17*). The frequency of thymic MHC class II^+^ cells expressing a given TRA correlates with the mechanism of T cell tolerance (*18*). A subset of mTEC^hi^ cells express autoimmune regulator (Aire), which drives the transcription of numerous TRA genes and is critical for establishment of T cell tolerance (*17*). Loss of human AIRE causes APECED (autoimmune polyendocrinopathy-candidiasis-ectodermal dystrophy), a rare, complex disease leading to tissue-specific autoimmune issues that differ widely between individuals.

We have been interested in how vitamin D signaling contributes to critical thymic events controlling establishment of T cell tolerance. Hormonally active 1,25-dihydroxyvitamin D (1,25D) is produced by consecutive hydroxylations, consisting of largely hepatic 25-hydroxylation, followed by 1α-hydroxylation catalyzed by the enzyme Cyp27b1 (*19*). 1,25D activates the vitamin D receptor (Vdr), a member of the nuclear receptor family and ligand-regulated transcription factor. We found that the Vdr and Cyp27b1 are expressed in both hematopoietic and stromal compartments of the thymus, including mTECs, and that, moreover, Aire functions as a transcriptional cofactor of the hormone-bound Vdr (*20*). These findings link vitamin D signaling to transcriptional events required for central tolerance. They also provide a molecular basis for clinical studies linking vitamin D deficiency to increased risk of development of autoimmune disorders (*21*–*26*).

Here, we investigated the effects of loss of 1,25D signaling on thymic morphogenesis as well as the differentiation and function of TECs using a *Cyp27b1* knockout (CypKO) mouse model. Thymi of null animals displayed a combination of markedly reduced cellularity, which accelerated with age, and skewed mTEC differentiation, favoring Ccl21^+^ Aire^−^ over Aire^+^ cells. This coincided with reduced Aire expression, impaired TRA gene transcription in CypKO thymi, and a reduction in markers of T cell negative selection. Moreover, single-cell gene expression profiling of TEC populations revealed gene expression patterns in null animals consistent with premature thymic aging, characterized by impaired expression of key thymic longevity factors. On the basis of these findings, we conclude that 1,25D signaling is necessary for normal thymic development, longevity, and mTEC differentiation and function necessary for establishment of central tolerance.

## RESULTS

### Reduced cellularity and altered conventional T cell development in CypKO thymi

**Fig. 1.**
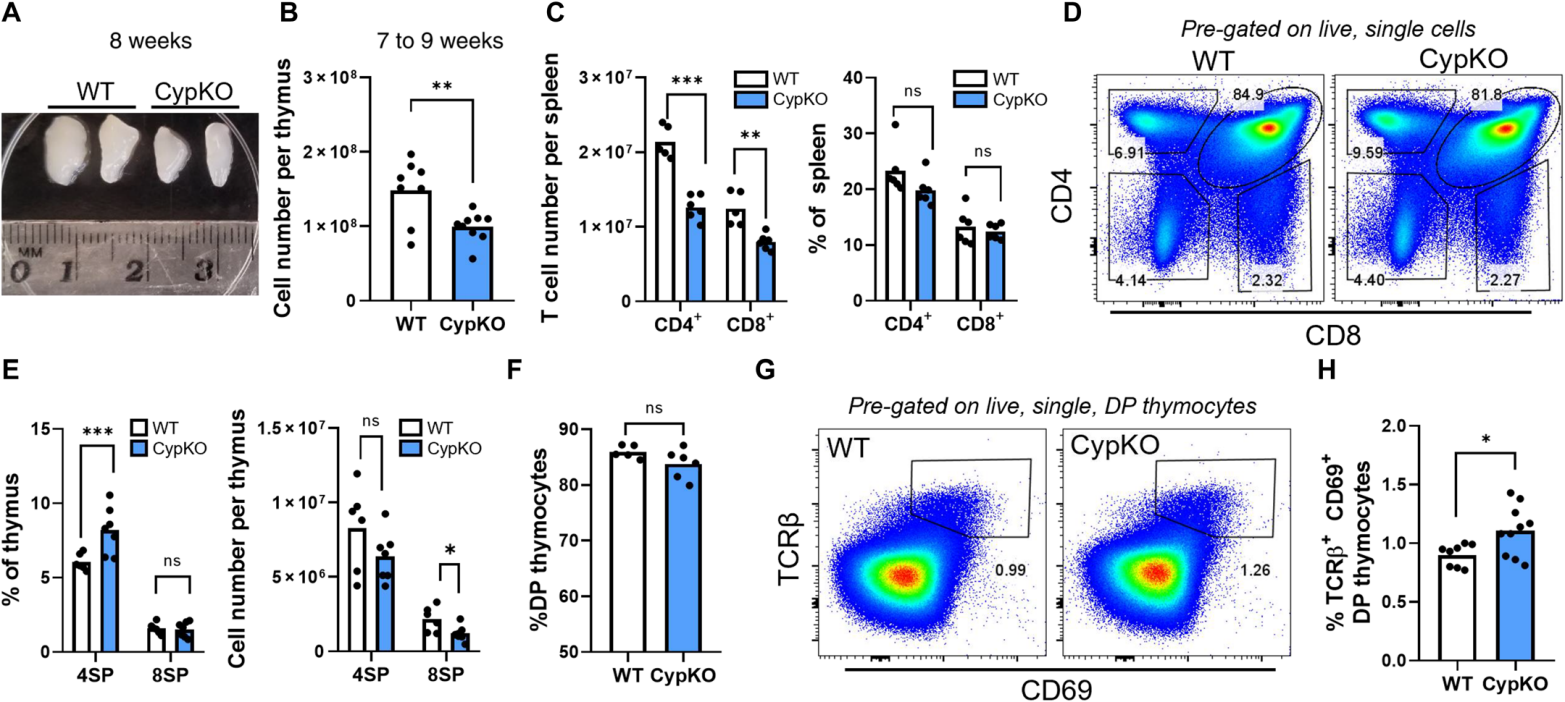
Reduced thymic cellularity and altered T cell development in CypKO mice. (**A**) Representative picture of individual thymic lobes from two WT and CypKO 8-week-old female mice. (**B**) Total thymic cell number in 7- to 9-week-old mice. (**C**) Numbers of total CD4^+^ and CD8^+^ splenic T cells (left) and frequencies (right). (**D** to **F**) Representative flow cytometry plots of thymocyte development (D), quantification of single-positive (SP) thymocyte frequencies [(E), left] and numbers [(E), right)], and DP thymocyte frequencies (F). (**G** and **H**) Representative flow cytometry plots of post-selection DP thymocyte frequencies (G) and summary data (H). All experiments were performed with age-matched (male and female) 7- to 9-week-old mice. Each dot represents an individual mouse. ns, not significant. Statistics: Unpaired parametric *t* tests. *P < 0.05; **P < 0.01; ***P < 0.001.

CypKO mice were originally generated as a model of 1,25D-dependent rickets, a disease of bone growth arising from inadequate dietary calcium absorption. However, calcium homeostasis can be normalized by raising CypKO mice on a calcium and lactose rescue diet (*27*). Thymi from 4-week-old wild-type (WT) and CypKO animals raised on a rescue diet were similar in size (fig. S1, A and B). In contrast, those of 7- to 9-week-old CypKO mice were significantly smaller than controls (Fig. 1A), corresponding to an ~35% reduction in total cellularity (Fig. 1B). Reduced thymic size was accompanied by decreased splenic CD4^+^ and CD8^+^ T cell numbers in CypKO animals (Fig. 1C), consistent with systemic lymphopenia. The frequency of splenic naïve CD4^+^, but not CD8^+^, T cells was marginally reduced in CypKO animals (fig. S1C), whereas the proportion of FoxP3^+^ T_regs_ was not significantly different (fig. S1D).

Thymocyte development in CypKO mice was grossly normal, with unaltered frequencies of γδ T cells (fig. S1, E and F) and NK1.1^+^ αGalCer tetramer^−^ natural killer (NK) cells (fig. S1, G and H). In agreement with previous data, we observed decreased frequencies of αGalCer tetramer^+^ invariant NK T (iNKT) cells (fig. S1, I and J) (*28*). For αβ T cells, frequencies of CD4^+^ single-positive (4SP) thymocytes were elevated, whereas those of double-negative (DN), double-positive (DP), and CD8^+^ single-positive (8SP) thymocytes were unaltered (Fig. 1, D to F). This is in contrast to a previous study that found that 4SP thymocytes were unaltered in CypKO thymi (*29*). Among 4SP thymocytes, the proportion of FoxP3^+^ CD25^+^ T_regs_ was unchanged (fig. S1, K and L). The increased proportion of 4SP thymocytes in CypKO thymi suggested potential alterations in thymic selection. However, the expression of T cell receptor (TCR) signaling molecules up-regulated on positively selected thymocytes, including CD5, CD69, and TCRβ, was not significantly different in CypKO post-selection DP thymocytes (fig. S1M), suggesting that CypKO thymocytes do not experience stronger TCR signaling during thymic selection. In contrast, post-selection DP thymocytes were modestly increased in CypKO thymi (Fig. 1, G and H), suggesting enhanced positive selection or decreased negative selection.

### Diminished APC populations and altered mTEC differentiation in CypKO thymi

**Fig. 2.**
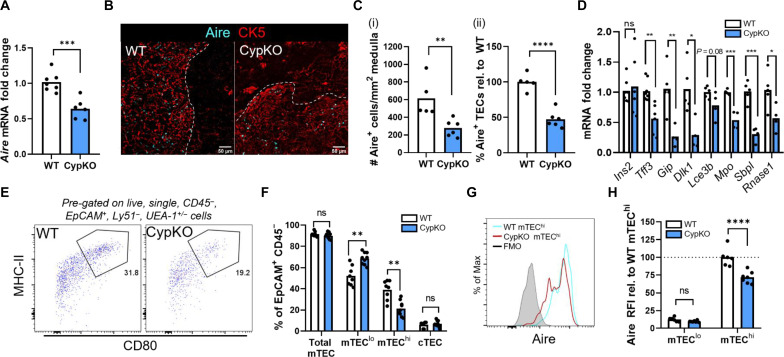
Impaired mTEC differentiation and Aire expression in CypKO mice. (**A**) qPCR detection of Aire mRNA expression in WT versus CypKO mice. (**B** and **C**) Zoomed-in IF microscopy images showing Aire ^+^ cells in WT versus CypKO thymi (B) and quantification from the entire thymic cross sections (C) (white dashed line denotes the cortico-medullary boundary). (**D**) qPCR detection of Aire-dependent TRA gene expression in WT versus CypKO thymi. (**E**) Representative flow cytometry plots of mTEC^hi^ cells (MHC-II^hi^ CD80^+^) in WT versus CypKO thymi. (**F**) Quantification of cTEC and mTEC frequencies in WT versus CypKO samples by flow cytometry. (**G**) Representative flow cytometry plot of Aire expression in WT versus CypKO mTEC^hi^ cells. (**H**) Quantification of Aire expression in mTEC^lo^ and mTEC^hi^ cells, relative to the average MFI of Aire in WT mTEC^hi^ cells. All experiments were performed with age-matched (male and female) 8- to 12-week-old mice. Each dot represents an individual mouse. Statistics: Unpaired parametric *t* tests. *P < 0.05; **P < 0.01; ***P < 0.001; ****P < 0.0001.

mTECs are critically important for the negative selection of overly self-reactive thymocytes. We previously found that 1,25D signaling induced Aire expression in mTECs in cultured mouse thymic slices (*20*). Consistent with these findings, there was a 40% reduction in *Aire* mRNA in CypKO thymi (Fig. 2A). Unlike in WT thymi, the medulla in CypKO mice could not be clearly defined by cytokeratin 5 (CK5) expression (Fig. 2B). However, visualization of Aire along with mTEC and medullary marker *Ulex europaeus* agglutinin 1 (UEA-1) revealed tight costaining in both WT and CypKO samples (fig. S2A).

Furthermore, CD11c^+^ dendritic cells (DCs) were concentrated in Aire^+^ regions in both WT and CypKO thymi (fig. S2B). In addition, a decreased density of 4′,6-diamidino-2-phenylindole (DAPI) staining, characteristic of the thymic medulla, could be observed in both samples (fig. S2B). Together, these data indicate that CypKO mice have discernible medullary structures as defined by Aire and other markers. We quantified the density of Aire^+^ cells in medullary regions and found that they were reduced by ~50% in null thymi as quantified by immunofluorescence (IF) microscopy (Fig. 2, B and C). Furthermore, the expression of multiple Aire-dependent TRA mRNAs was variably reduced in CypKO samples (Fig. 2D). These results corresponded with a depletion of mature Aire^+^ mTECs (mTEC^hi^) with a proportional increase in mTEC^lo^ populations (Fig. 2, E and F, and fig. S2C), whereas cTEC frequencies were unaltered. Notably, Aire relative fluorescence intensity (RFI; relative to average WT Aire expression) was reduced by ~30% in CypKO versus control mTEC^hi^ cells (Fig. 2, G and H).

Phenotypic differences between Cyp27b1-deficient and Vdr-deficient mice and humans (e.g., alopecia in the latter only) (*27*, *30*, *31*) have been documented and attributed to 1,25D-independent functions of the Vdr. Therefore, we used *Vdr*-null (VdrKO) mice to complement our findings. As in CypKO mice, VdrKO thymi in 8- to 10-week-old, but not 5-week-old, animals had reduced cellularity compared to littermate controls (fig. S3A), and *Aire* mRNA was reduced by ~30% in VdrKO samples (fig. S3B). Loss of the Vdr had a variable effect on TRA genes, with expression of only a subset being reduced (fig. S3C). The mTEC phenotype in VdrKO mice as assessed by flow cytometry was consistent with that in CypKO animals, with reduced frequencies of mTEC^hi^ cells (fig. S3, D and E) and reduced mean Aire expression (fig. S3, F and G). Together, our results show that Aire expression is diminished, mTEC^hi^ differentiation is impaired, and Aire expression is reduced within differentiated cells in the absence of vitamin D signaling.

In addition to TEC populations, hematopoietic antigen-presenting cells (APCs) including DCs and B cells contribute to negative selection. In CypKO mice, DC frequencies were unaltered (fig. S4, A and B), whereas those of B cells were halved (fig. S4, C and D). Thymic B cells express Aire and play a nonredundant role in thymocyte negative selection (*32*). However, unlike mTEC^hi^ cells, Aire expression was unaffected in CypKO thymic B cells (fig. S4, E and F). Moreover, there was no evidence of a failure to commit to tissue residency as measured by frequencies of committed thymic IgM^+^ IgD^−^ and IgM^−^ IgD^−^ subpopulations (fig. S4, G and H), which would be indicative of a potentially systemic defect. B cell numbers were also halved in CypKO spleens (fig. S4I). Collectively, these data show that the frequency of the APCs involved in the negative selection of thymocytes, including Aire^+^ mTECs and B cells, is diminished and that *Aire* and Aire-dependent TRA gene expression in CypKO mTECs is reduced.

### Decreased thymocyte apoptosis consistent with impaired negative selection in CypKO thymi

**Fig. 3.**
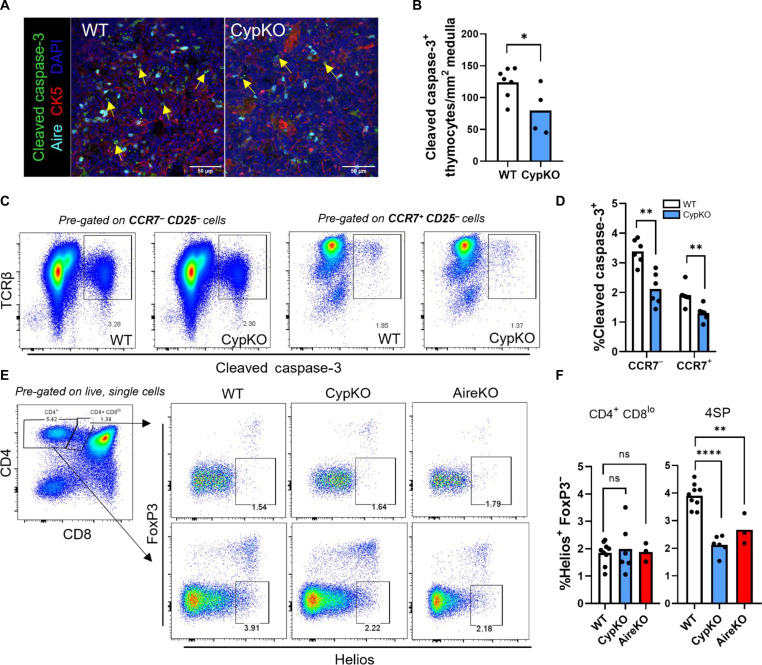
Decreased thymocyte apoptosis in CypKO mice. (**A** and **B**) IF microscopy images of cleaved caspase-3 staining in the thymic medulla in small CK5^−^ cells (A) and quantification of positive cell density (B) in WT versus CypKO from the entire thymic cross sections. (**C** and **D**) Representative flow cytometry plots of cleaved caspase-3^+^ cells in WT versus CypKO CCR7^−^ CD25^−^ (left) or CCR7^+^ CD25^−^ thymocytes (right) (C) and quantification of cleaved caspase-3^+^ thymocytes (D). (**E** and **F**) Representative flow cytometry plots of Helios^+^ FoxP3^−^ CD4^+^ CD8^lo^ (top) or 4SP (bottom) thymocytes in WT versus CypKO or Aire KO thymi (E) and summary data (F). All experiments were performed with age-matched (male and female) 8- to 12-week-old mice. Each dot represents an individual mouse. Statistics: Unpaired parametric *t* tests. *P < 0.05; **P < 0.01; ****P < 0.0001.

Next, we investigated the potential for altered negative selection in CypKO thymi by first quantifying the number of apoptotic thymocytes in the thymic medulla by IF. The number of cleaved caspase-3^+^ thymocytes was reduced in the medulla and cortex of CypKO mice (Fig. 3, A and B, and fig. S5, A to C). Further analysis by flow cytometry corroborated this finding and showed a significant decrease in apoptotic medullary (CCR7^+^) and cortical (CCR7^−^) CypKO thymocytes (Fig. 3, C and D, and fig. S5D). Apoptotic DP thymocytes with up-regulated TCR signaling molecules (“signaled” thymocytes) such as CD5 or TCRβ have been shown to be undergoing negative selection, whereas apoptotic CD5^−^ TCRβ^lo^ DP thymocytes are undergoing death by neglect (“not signaled” thymocytes) (*33*). The frequency of cleaved caspase-3^+^ cells of signaled and not signaled thymocytes was equally diminished in CypKO mice (fig. S5, E and F), indicating a general thymocyte survival phenotype in the absence of 1,25D signaling. We then investigated the expression of the transcription factor Helios, which is up-regulated in negatively selected thymocytes and is a marker of highly autoreactive FoxP3^−^ cells (*34*, *35*). There was a 40% reduction in the frequency of Helios^+^ FoxP3^−^ 4SP thymocytes but not CD4^+^ CD8^lo^ thymocytes in CypKO mice (Fig. 3, E and F). This reduction was comparable to that observed in the thymi of Aire KO mice (Fig. 3, E and F), which are defective in negative selection (*36*).

**Fig. 4.**
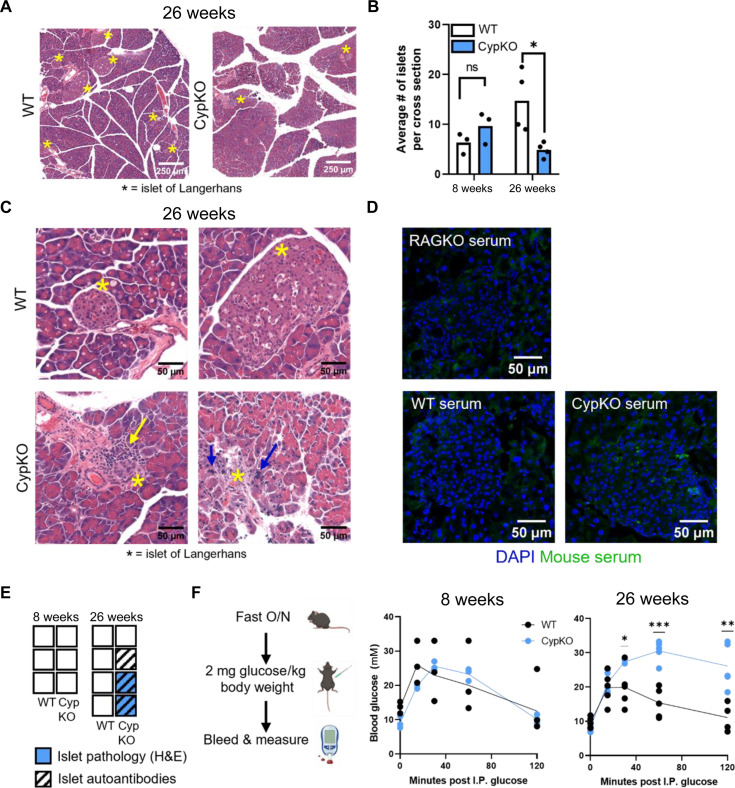
Evidence of disrupted pancreatic function and islet pathology in aged CypKO mice. (**A**) Representative H&E images of pancreases from 26-week-old WT and CypKO mice. Islets are marked with an asterisk. (**B**) Quantification of the average number of islets from duplicate whole-tissue cross section scans from 8- and 26-week-old WT and CypKO mice. (**C**) High-magnification images of WT or CypKO islets from duplicate mice. Immune cell infiltration is indicated with a yellow arrow, and pycnotic nuclei are indicated with blue arrows. (**D**) Representative images of autoantibody staining of islets with 26-week-old CypKO, WT, or Rag1KO serum samples. (**E**) Summary of indicated phenotypes in each mouse. Empty boxes indicate that no pathology or autoantibody staining was observed. (**F**) Experimental design for the glucose tolerance test (left) and blood glucose levels in 8- or 26-week-old WT versus CypKO mice after intraperitoneal (I.P.) glucose administration. All experiments were performed with age-matched (male or female) 8- or 26-week-old mice, except for histology for aged animals, which was performed on male mice exclusively. O/N, overnight. Statistics: Each dot represents an individual mouse. Unpaired parametric *t* tests. *P < 0.05; **P < 0.01; ***P < 0.001.

The thymic phenotype in CypKO mice is consistent with that of impaired negative selection. Therefore, we further investigated potential functional consequences by assessing the presence of spontaneous autoimmune phenotypes in aged CypKO versus WT mice. Previous studies have shown that the penetrance and severity of immune infiltrates and organ-specific autoantibodies in Aire KO mice are highly variable and increase with animal age to at least 20 to 30 weeks (*37*–*39*). Thus, 26-week-old WT and CypKO mice were assessed for the presence of immune infiltrates and organ-specific autoantibodies by histology and IF microscopy, respectively. Evidence of mild inflammation could be observed in the lung alveoli, salivary gland, and supra-adrenal fat, whereas no histological abnormalities were seen in the small intestine, prostate, thyroid, brain, liver, or kidney of aged CypKO mice (fig. S6A). Histological analysis of the pancreas showed that aged, but not young (8 weeks), CypKO mice contained significantly fewer islets of Langerhans (islets) than WT controls (Fig. 4, A and B). While islets found in CypKO pancreases were mostly normal, there was evidence of immune cell infiltrates or necrosis in the islets of two of four mice (Fig. 4C). While the diminished number of islets in CypKO animals may be explained by a protective effect of vitamin D on β cell survival (*40*), it cannot explain the presence of the observed pathologies. Moreover, autoantibody staining specific to islet cells was observed in three of four aged CypKO mice (Fig. 4, D and E). No such pathologies were observed in islets of aged WT controls. Autoantibody staining was also observed in the stomach (two of four animals) but not retina, salivary gland, or lung of CypKO mice (fig. S6, B to E).

The histological data suggested that pancreatic endocrine function may be impaired in CypKO mice in an age-dependent manner. We probed this further by performing a glucose tolerance test and found that aged, but not young, CypKO mice displayed severely compromised glucose clearance (Fig. 4F). Together, these data provide evidence for an organ-specific immune phenotype in CypKO mice. The amplitude of the tissue infiltration observed was not unexpected, given data from Aire-deficient mice, which display a partially penetrant autoimmune phenotype (*37*–*39*).

### Single-cell RNA sequencing of TEC populations reveals disrupted mTEC differentiation in CypKO mice

**Fig. 5.**
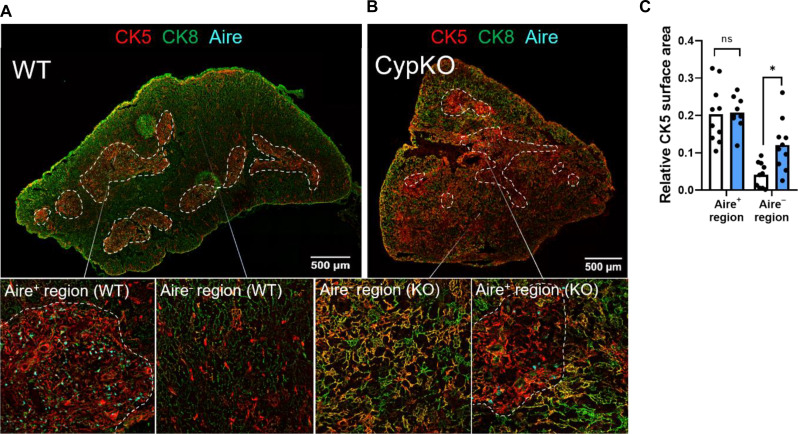
Disorganized thymic architecture in CypKO mice. (**A**) Representative images of a WT thymic cross section with Aire staining restricted to areas of concentrated CK5 staining (medulla). (**B**) Representative images of a CypKO thymic cross section showing disseminated CK5 staining in Aire^−^ regions. (**C**) Quantification of CK5 staining in the medulla (defined as Aire^+^ regions) and cortex (defined as Aire^−^ regions). All experiments were performed with age-matched (male and female) 8- to 12-week-old mice. Each dot represents an individual mouse. Statistics: Unpaired parametric *t* tests. *P < 0.05.

The data suggest that negative selection in CypKO thymi is impaired and that this may be due, at least in part, to defects in mTEC differentiation and Aire expression. Proper organization of the thymic cortex and medulla is critical for thymocyte selection and mTEC differentiation as shown in mice deficient in various factors that regulate mTEC differentiation, including *Relb* (*41*), *Nik* (*42*), *Ikka* (*43*), *Traf6* (*44*), *Ltbr* (*45*), and *Nfkb2* (*46*). The medulla in CypKO animals could be defined based on regions of Aire expression, concentration of DCs, and diminished DAPI density (Fig. 2). In WT thymi, CK5 staining overlaps tightly with medullary Aire^+^ regions. However, costaining with CK5 and cytokeratin 8 (CK8), a pan-TEC marker, revealed a significantly increased number of CK5/8 double-positive cells in the CypKO cortex, extending well beyond the domains containing Aire^+^ cells (Fig. 5, A to C), consistent with disrupted TEC differentiation in CypKO animals.

**Fig. 6.**
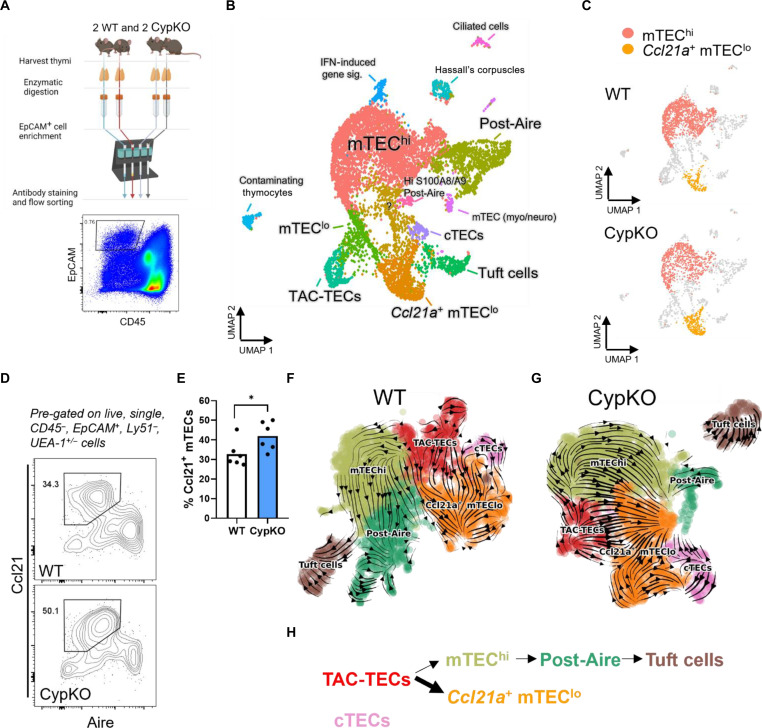
Divergent mTEC differentiation in CypKO mice. (**A**) Schematic of single-cell RNA-seq sample preparation from duplicate littermate mice. (**B**) Cluster annotation of integrated Seurat object containing all four samples, based on differential gene expression. IFN, interferon. (**C**) Comparison of WT and CypKO clusters densities; mTEC^hi^ and Ccl21a^+^ mTEC^lo^ are highlighted. (**D** and **E**) Validation of increased Ccl21^+^ mTECs in CypKO thymi by flow cytometry (D) and summary data (E). Each dot represents an individual mouse (age-matched, male and female). (**F** and **G**) RNA velocity plots of WT (F) and CypKO (G) samples; arrows point toward the direction of predicted differentiation based on RNA splicing dynamics. (**H**) Schematic of altered mTEC differentiation in CypKO mice. Statistics: Unpaired parametric *t* tests. *P < 0.05.

To investigate TEC development in *Cyp27b1*-deficient mice further, we sorted total TECs (CD45^−^ EpCAM^+^) from duplicate CypKO and WT littermates and performed single-cell RNA sequencing (RNA-seq) (Fig. 6A). A similar decrease in *Aire* and TRA gene expression was seen in CypKO animals from F2 littermates compared to mixed background CypKO mice (Fig. 2, C and D, and fig. S7A). Eighteen clusters were annotated (Fig. 6B) based on differentially expressed genes (DEGs; see table S1 for top 25 DEGs per cluster and fig. S7B for heatmap). mTEC^lo^, mTEC^hi^ and post-Aire cells predominated, along with smaller populations of cTECs and Tuft cells (*5*, *47*, *48*). A cluster enriched for S phase and G_2_-M markers was consistent with so-called transient amplifying cells (TAC-TECs) (*47*). Several minor clusters included those with markers for ciliated cells (*49*), Hassall’s corpuscles, as well as populations with markers expressed in mTEC neuro and mTEC myo cells previously identified in human thymi (Fig. 6B and table S1) (*50*, *51*).

As expected, the number of mTEC^hi^ cells was reduced in CypKO samples, whereas *Ccl21a^+^* cells were enriched (Fig. 6C and fig. S7, C and D). The elevated *Ccl21a* expression in CypKO thymi was validated by reverse transcription quantitative polymerase chain reaction (RT-qPCR) (fig. S7E) analysis of total thymic samples and flow cytometry (Fig. 6, D and E). Consistent with previous works (*5*, *49*), these cells were also enriched for expression of *Krt5*, *Il7*, *Lifr*, and *Pdpn* (fig. S8, A to F). Ccl21^+^ mTECs have been proposed to be precursors of mature Aire^+^ cells (*52*). However, a study has suggested that they represent a distinct differentiation path from *Aire*^+^ cells, with TAC-TECs as the initiation point for both (*47*). To probe this further, we used RNA velocity analysis, which incorporates information about spliced versus unspliced RNAs in single cells to predict transitions in cell state and estimate differentiation trajectories (*53*). After preprocessing with scanpy, clusters for new uniform manifold approximation and projection (UMAP) plots were annotated based on DEGs; minor clusters and unrelated cells (e.g., thymocytes) were removed. Velocity analysis using scVelo (*54*) of WT (Fig. 6F and fig. S9, A and B) samples was consistent with the bifurcation model, where *Ccl21a*^+^ mTECs do not appear to differentiate into mTEC^hi^ cells. Trajectories were similar in CypKO samples but with an increased density of arrows leading from TAC-TECs toward *Ccl21a*^+^ mTECs (Fig. 6, G and H, and fig. S9, C and D). Bifurcation was additionally corroborated by pseudotime analysis using Monocle 3 (fig. S9, C and D). Thus, these data suggest that, instead of impaired mTEC^lo^-mTEC^hi^ differentiation in the absence of 1,25D, differentiation is skewed toward Ccl21^+^ Aire^−^ cells at the expense of Aire^+^ cells.

### Premature aging of CypKO TEC populations

**Fig. 7.**
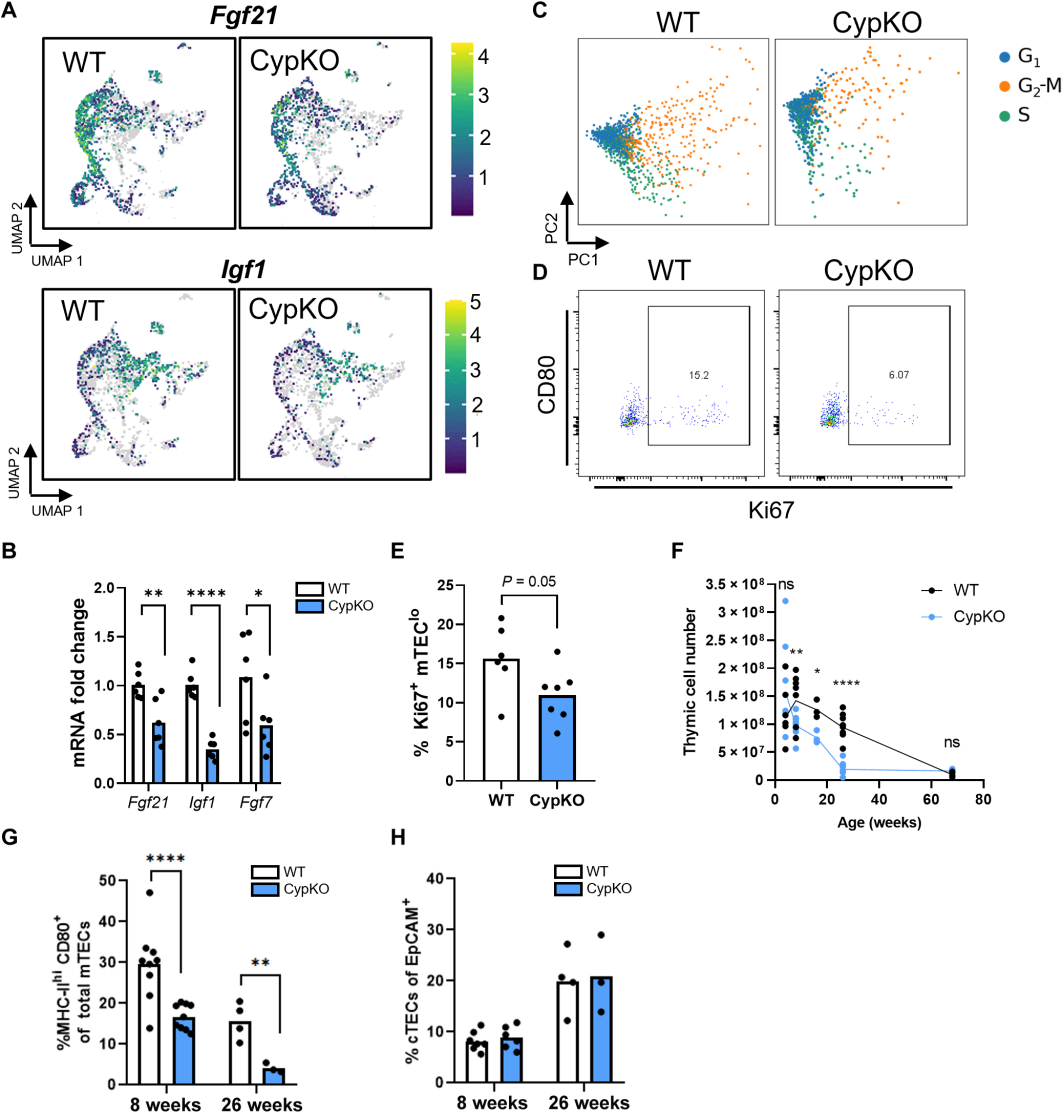
Accelerated thymic involution in CypKO mice. (**A**) UMAPs indicating decreased expression of pro-longevity factors *Fgf21*, *Fgf7*, and *Igf1* in CypKO TECs. (**B**) Validation of thymic involution related gene expression. (**C**) Cell cycle phase scoring for representative WT and CypKO TECs. (**D** and **E**) Flow cytometry analysis of Ki67^+^ in mTEC^lo^ cells. (**F**) Total thymic cell counts from young to old mice showing accelerated involution in CypKO samples. (**G**) Frequency of mTEC^hi^ cells of total mTECs. (**H**) Frequency of cTECs of total TECs. All experiments were repeated at least twice with age-matched (male and female) mice. Each dot represents an individual mouse. Statistics: Unpaired parametric *t* tests. *P < 0.05; **P < 0.01; ****P < 0.0001.

TEC differentiation is regulated by multiple inputs, including intracellular signaling and cross-talk with thymocytes and other stromal cells (*55*). We used Ingenuity Pathway Analysis (IPA) to better understand the dysregulated signals in CypKO TECs. Notably, VDR/retinoid X receptor activation was among the top 5 altered canonical pathways in CypKO versus control TECs (fig. S10A). After filtering for thymic datasets and ranking by activation *Z* score, the most similar dataset to ours compared lymphocytic choriomeningitis virus (LCMV) infection to control thymi (*Z* score = 32.67%), which is noteworthy as LCMV infection is characterized by severe thymic atrophy and impaired negative selection (*56*). The second most similar dataset to ours was a comparison between middle-aged and young mice (*Z* score = 24.28%) (fig. S10, B and C). Aging is associated with decreased thymic cellularity and impaired production of naïve T cells, primarily due to defects in the aged thymic stroma (*3*). We found that upstream regulators induced during thymic involution were predicted to be activated in CypKO TECs (fig. S10, B and C). Furthermore, there was decreased expression in CypKO thymi of several loci encoding proteins that contribute to thymic longevity, notably fibroblast growth factor 21 (*Fgf21*), fibroblast growth factor 7 (*Fgf7*), and insulin-like growth factor 1 (*Igf1*) (*57*–*60*). RT-qPCR validation of these genes in sorted TECs corroborated the reduction in gene expression by single-cell RNA-seq (Fig. 7, A and B). Furthermore, expression of *Crip3*, whose ablation is associated with decreased thymic cellularity (*61*), was reduced in CypKO samples (fig. S10D). A key hallmark of thymic involution is impaired TEC proliferation (*3*); *Mki67*-expressing cells were reduced in CypKO samples (fig. S10E), and there was diminished expression of G_1_-S and G_2_-M phase-specific genes (Fig. 7C). The reduced number of Ki67^+^ mTEC^lo^ cells was validated by flow cytometry (Fig. 7, D and E), consistent with attenuated TEC proliferation in CypKO thymi.

The above changes were largely consistent with those in three independent TEC gene expression profiling datasets from young and aged mice (fig. S11, A to C), the only discrepancy being an increase in *Igf1* expression observed in two of the three datasets. Furthermore, markers of “intertypical” TECs, including *Ccl21a*, *Krt5*, *Pdpn*, and *Lifr*, increased with aging in these datasets (fig. S11, A to C). Cluster annotation of integrated single-cell RNA-seq data from 1- to 52-week-old mouse TECs revealed an enrichment of the *Ccl21a*^+^ cluster with age (fig. S11, D and E). Thus, perturbations in gene expression in CypKO TECs are consistent those occurring during thymic aging. As thymic atrophy in WT mice begins at 3 to 4 weeks and continues for the first year of life, we compared control and CypKO thymi (male and female) and found that cellularity in CypKO animals steeply declined by 26 weeks of age, compared to an 18% reduction in age-matched and sex-matched controls (Fig. 7F). Furthermore, mTEC^hi^ cells were disproportionally depleted in CypKO thymi with age (Fig. 7, G and H). We conclude that loss of 1,25D impairs normal TEC differentiation and negative selection of thymocytes and accelerates age-associated thymic involution.

## DISCUSSION

Previous studies have identified a role for vitamin D signaling in the suppression of peripheral proinflammatory T cell responses (*62*). This is consistent with links between vitamin D deficiency and increased incidence of autoimmune conditions such as type 1 diabetes (T1D) and multiple sclerosis (MS) (*21*, *25*). However, the potential prophylactic and therapeutic benefit of vitamin D supplementation in adults remains unclear due to conflicting findings (*21*). In contrast, a study with children revealed clear associations between vitamin D status and T1D risk (*63*). Because the naïve T cell pool is sustained by thymic output in the first 20 years of life (*64*), these studies suggest that vitamin D status may be especially relevant in early life during the period where thymic output is at its highest. In this regard, prospective and cross-sectional studies have provided evidence linking gestational or neonatal vitamin D deficiency in humans to reduced thymic perimeter and transverse diameter (*65*, *66*).

Here, we showed that 1,25D signaling is essential for normal Aire^+^ TEC maturation, TRA gene expression, and thymic tissue development. Our findings link clinical observations to an in vivo thymic phenotype and provide a molecular basis for the potential role of vitamin D signaling in thymic homeostasis during childhood and infancy. Negative selection occurs in two waves: first in the cortex through interactions of developing thymocytes with DCs, followed by a second wave in the medulla through interactions with mTECs, DCs, and B cells (*15*, *35*). B cell, but not DC, frequencies were reduced in CypKO thymi. Given their respective roles in negative selection, it would be of interest to further quantify the contribution of the loss of vitamin D signaling in thymic hematopoietic cells on the central tolerance phenotype. Cortical and medullary negative selection of thymocytes can be tracked by flow cytometry by gating on CD4^+^ CD8^low^ (cortical, CCR7^−/low^) and 4SP (medullary, CCR7^+^) cells. The transcription factor Helios serves as a readout of highly autoreactive T cells in the thymus (*35*) and is up-regulated on negatively selected cells and down-regulated on positively selected cells (*35*). In addition to impaired Aire^+^ mTEC development, we observed decreased levels Helios^+^ FoxP3^−^ 4SP thymocytes in CypKO mice. In agreement with previous data (*34*), Helios^+^ FoxP3^−^ 4SP thymocytes were reduced in AireKO mice and, to a similar extent, CypKO mice. Helios expression in cortical CD4^+^ CD8^low^ thymocytes was unaltered in both Cyp27b1-deficient and Aire-deficient thymi. This suggests that decreased TRA transcription in both AireKO and CypKO does not affect cortical negative selection. While complete abrogation of AIRE results in a marked autoimmune phenotype, the effects of vitamin D deficiency in humans on autoimmune phenotypes are more subtle. In this regard, Aire function is attenuated but not eliminated in CypKO animals.

VdrKO mice had a similar, albeit somewhat attenuated, TEC phenotype, characterized by decreased *Aire*, TRA gene expression, and mTEC^hi^ differentiation as well as accelerated age-dependent thymic involution. The difference in the degree of the TEC phenotype between the two KO models is not unexpected, given previous reports of phenotypic differences between these null mice (*29*, *67*). Notably, loss of the VDR, but not CYP27B1, in mice and humans leads to alopecia totalis (*27*, *30*, *68*, *69*). This is due to 1,25D-independent interactions between the Vdr with the corepressor Hairless (Hr) in epidermal keratinocytes (*70*). Phenotypic differences between the two models have also been observed under immune-related conditions. 1,25D-deficiency augments incidence and disease severity in mouse models of T1D and MS (*71*–*73*). Nonobese diabetic mice maintained on a vitamin D deficient diet in early life have a higher incidence of T1D later in life (*72*). However, disease activity is unaltered or diminished in Vdr-deficient mice for both MS and T1D models (*74*, *75*). Currently, the signals that drive these phenotypic differences are poorly understood.

Our data showed that there was mild but widespread inflammation in aged CypKO mice, with evidence of immune infiltrates and autoantibodies targeting the pancreas. This is in agreement with data showing that the presence of 1,25D is protective in autoimmune diseases (*76*). Much is known about the function of vitamin D signaling in mature T cells, which suppresses CD4^+^ and CD8^+^ T cell proliferation (*77*, *78*) and favors T_H_2 (T helper 2 cell) and T_reg_ function while suppressing proinflammatory cytokine production (*79*–*83*). In conclusion, while we cannot rule out the potential contribution of regulation by vitamin D of peripheral tolerance on the autoimmune phenotype in CypKO mice, our data provide strong evidence for a role of vitamin D signaling in the establishment of central tolerance.

In addition to the impairment of negative selection in CypKO thymi, we observed notable changes in tissue organization characterized by an outgrowth of CK5 and CK8 expressing TECs. A previous report indicates that these double-positive cells are TEC progenitors (*84*). Our single-cell RNA-seq results corroborated the increased presence of a CK5 (*Krt5*) expressing population that also expressed *Ccl21a* and markers of junctional TECs (jTECs) such as Pdpn (*52*). However, due to differences in tissue localization of Ccl21^+^, Pdpn^+^, and CK5^+^ CK8^+^ populations, it is likely that this *Ccl21a*^+^ cluster represents multiple populations with similar gene expression. This is consistent with findings that *Ccl21a*^+^ *Krt5*^+^ intertypical TECs have characteristics of heterogeneous TEC populations, including jTECs, TPA^lo^, and Sca-1^+^ TECs, and have mature mTEC precursor potential (*85*). However, if Ccl21^+^ TECs are precursors of mature Aire^+^ mTECs, representing a block in development at this stage in CypKO mice, or represent a distinct terminally differentiated population is disputed (*47*, *49*, *52*). Our findings in CypKO mice are consistent with the branching differentiation model, suggesting that 1,25D promotes Aire^+^ TEC differentiation, whereas, in its absence, *Ccl21a*^+^ TEC differentiation is favored.

Last, we found premature thymic involution and a gene expression profile consistent with accelerated thymic aging in CypKO mice. Thymic involution is characterized by reduced output of naïve T cells and progressive atrophy and structural changes (*86*), which were apparent as early as 8 weeks in CypKO thymi. These thymic changes contribute to a peripheral phenotype of immunosenescence where the host loses the ability to fight new infections and clear tumors due to reductions in T cell repertoire diversity (*86*). Thymic involution arises largely from defects in the thymic stromal niche causing changes in the thymic microenvironment (*86*). In CypKO mice, thymic cellularity was markedly diminished by 26 weeks, whereas reductions in WT thymi were modest (~18%). We also found that CypKO thymi displayed other hallmarks of aging such as reduced TEC proliferation and distorted cortical-medullary boundaries (*87*). Furthermore, the expression of *Fgf21*, *Fgf7*, and *Igf1* was down-regulated in CypKO TECs. These genes encode proteins that have distinct roles in positively regulating thymic cellularity, naïve T cell output, TEC numbers, and thymic precursor proliferation (*57*, *58*, *60*). Notably, these genes are down-regulated in aging thymi and are antithymic involution factors (*3*). Both CypKO and VdrKO mice had unaltered thymic cellularity at 4 to 5 weeks of age, which then rapidly degenerated with respect to WT controls. This coincides with the peak of thymic development in mice at 4 weeks, which is followed by the early stages of thymic involution (*85*). The expression of the Vdr and Cyp27b1 in thymic stromal and hematopoietic compartments is consistent with autocrine production of 1,25D contributing to proper TEC differentiation and thymic morphogenesis. Data from our previous study showed that administration of 1,25D to mouse thymic slices is sufficient to drive *Aire* expression and increase the number of Aire^+^ cells (*20*). Therefore, it would be of interest to determine if in vivo treatment with 1,25D could prevent or reverse the involution phenotype, the effects on TEC differentiation, and Aire expression in CypKO animals. In addition to determining the developmental window during which 1,25D treatment would potentially be most efficacious, careful consideration of the dose must be taken to prevent hypercalcemia in treated animals, which, in addition to the known effects of calcium on thymic size (*88*, *89*), may exert unknown effects on other aspects of thymic biology.

Collectively, the phenotype in CypKO mice is associated with accelerated thymic aging, characterized by decreased proliferating TECs and expression of genes encoding multiple prothymic cytokines that control TEC proliferation and homeostasis. Aire^+^ TEC differentiation was impaired in CypKO mice, whereas that of Ccl21^+^ TECs was enhanced and was associated with a phenotype consistent with defective negative selection. The expression of Cyp27b1 in both hematopoietic and stromal compartments, notably mTECs, strongly suggests that local, as opposed to systemic, production of 1,25D is critical for vitamin D signaling in the thymus. In future experiments, it will be important to determine the relative contributions of hematopoietic versus stromal production of 1,25D to thymic development and function using tissue-specific KOs.

## MATERIALS AND METHODS

### Study design

Our initial hypothesis was that vitamin D signaling may play a role in TEC differentiation and function, based on the expression of the Vdr and Cyp27b1 in TEC populations and the functional association of the Vdr and Aire (*20*). We used *Cyp27b1*-deficient mice, which cannot generate hormonal 1,25D. We compared thymic hematopoietic and stromal populations in CypKO and WT thymi by flow cytometry and IF microscopy. We observed a notable decrease in Aire^+^ mTECs and Aire expression in CypKO animals. Quantification of cells from tissue sections was performed both blinded and unblinded by multiple researchers. The abnormal distribution of the mTEC marker CK5 in CypKO thymi further supported altered mTEC differentiation. This was further investigated by single-cell RNA-seq on sorted total TECs from duplicate WT and CypKO mice. Analysis of this data was unblinded. “WT1” and “KO1” samples were used for UMAP plots, velocity analysis, cell cycle analysis, and generation of DEG tables per cluster and IPA because they presented a more typical phenotype of each respective group based on other experimental data (e.g., “KO2” had unusually reduced *Aire* expression). Descriptions of biological replicates are included in figure legends. Replicates were only excluded when technical errors were recorded. All experiments were independently repeated two to four times (with *n* = 2 to 5 biological replicates per experiment), except for histological analyses, which were performed once on samples from four mice per group.

### Mice

*Cyp27b1* KO mice were generated as previously described (*27*) on a mixed C57BL/6 and BALB/C background and housed in the McGill University Health Center Research Institute. WT C57BL/6J mice were used as WT controls. Differences in *Aire* and TRA gene expression between F2 littermates were consistent with the differences observed when mixed background CypKO mice were compared to C57BL/6J controls. VdrKO mice on a C57BL/6 background were purchased from Jax (Jax no. 004743). The line was maintained via heterozygous littermate crosses. VdrKO and CypKO littermate controls, as of weaning, were kept on a rescue diet (*27*) to normalize systemic Ca^2+^ levels, and breeders were given Ca^2+^-supplemented water to assist with fertility. RagKO mice were purchased from Jax (Jax no. 002216). AireKO mice were purchased from Jax (Jax no. 002216). All animal studies were approved by the McGill University Facility Animal Care Committee [protocol nos. 4628 (D.G.) and MCGL-10060 (H.J.M)].

### Flow cytometry

Up to 5 × 10^6^ cells were incubated with a fixable viability dye (Thermo Fisher Scientific, 65-0866-18) diluted in phosphate-buffered saline (PBS) at 4°C for 20 min in 96-well plates. Cells were stained in a fluorescence-activated cell sorting (FACS) buffer [PBS + 1% fetal bovine serum (FBS) and 2 mM EDTA] containing fluorophore-conjugated antibodies (Abs) and Fc block for 30 min at 4°C. For analysis of intracellular proteins, cells were fixed and permeabilized using the FoxP3 Fix/Perm Kit (Thermo Fisher Scientific) for 30 min at room temperature (RT), protected from light. Cells were then resuspended in a 1X Perm/Wash buffer containing fluorophore-conjugated Abs and incubated for 30 min at 4°C. Cells and UltraComp eBeads (Thermo Fisher Scientific) were used for compensation controls. Samples were acquired on an LSRFortessa (BD Biosciences) and analyzed with FlowJo (BD Biosciences).

### Antibodies and dyes

The antibodies and dyes used are as follows: CD19 (1D3), MHC-II (M5/114.15.2), CD69 (H1.2F3), EpCAM (G8.8), CD45 (30-F11), Ly51 (6C3), CD80 (16-10A1), Aire (5H12), CD25 (PC61.5), FoxP3 (FJK-16s), Helios (22F6), TCRγδ (eBioGL3), CD44 (IM7), CD62L (MEL-14), CD5 (53-7.3), immunoglobulin D (IgD) (11-26), immunoglobulin M (IgM) (11/41), CD3 (17A2), Fixable Viability Dye eFluor 450, goat anti-rabbit immunoglobulin G (IgG) AF488, goat anti-rat IgG AF546, and 4′,6-diamidino-2-phenylindole, dihydrochloride (DAPI) (all from Thermo Fisher Scientific); CD4 (GK1.5), CD8 (53-6.7), TCRβ (H57-597), CK5 (Poly19055), Fc block (S17011E), NK1.1 (PK136), and CD11c (N418) (all from BioLegend); *Ulex Europaeus* Agglutinin I (UEA-1) (Vector Laboratories); CK8 (TROMA-1) (Developmental Studies Hybridoma Bank); mCD1d/αGalCer tetramer (NIH Tetramer Core Facility); Ccl21 (59106) (Bio-Techne); Cleaved Caspase-3 (D175) (Cell Signaling Technology); CCR7 (4B12) (R&D Systems); and goat anti-mouse IgG-PE (sc-3738) (Santa Cruz Biotechnology).

### Cell sorting

TECs were enriched prior to sorting by positively selecting for EpCAM^+^ cells (Miltenyi Biotec, 130-105-958). Flow cytometry labeling was performed as described above. Total TECs (live, EpCAM^+^, and CD45^−^) were sorted with a BD FACSAria Fusion into the FACS buffer.

### IF microscopy

Samples were fixed with 4% paraformaldehyde (PFA) for 1 hour and incubated with sucrose (Bioshop) solutions (10 to 30%) for 8 to 12 hours each. Samples were embedded using base molds (Fisher Scientific) containing O.C.T. compound (Fisher Scientific), frozen on dry ice, and stored at −80°C. Five- to 10-μm sections were obtained with a Leica CM3050 S Cryostat and Superfrost Plus slides (Fisher Scientific). A hydrophobic barrier was applied with a pen (Millipore Sigma), and slides were blocked with PBS containing 2% bovine serum albumin (w/v), 0.3% Triton X-100, and 1% FBS (blocking buffer) for 1 hour at RT. Slides were incubated in a humidified chamber with primary Abs diluted in the blocking buffer overnight at 4°C, secondary Abs conjugated to fluorophores diluted in the blocking buffer for 1 hour at RT, fluorophore-conjugated primary Abs for 1 hour at RT, and DAPI diluted in PBS for 5 min at RT. Slides were washed with 100 μM Tris buffer between each step, mounted with Fluoromount 4G (Thermo Fisher Scientific) and coverslips (no. 1.5, Fisher Scientific), and imaged with a Zeiss LSM710 confocal microscope with 20X or 63X objective. Images were analyzed with FIJI (ImageJ). Serum for autoantibody experiments was collected by either cardiac puncture or tail vein bleed and kept at −20°C after centrifugation. Seven- to 12-μm tissue sections obtained from organs harvested from RagKO mice were incubated with serum from 26-week-old WT or CypKO mice, or RagKO and Aire KO sera, at 1:20 to 1:40 dilutions and incubated overnight at 4°C. For the remainder of the protocol, slides were treated as above [with anti-mouse secondary antibodies conjugated to phycoerythrin (PE)]. Autoantibody staining positivity was scored by seven participants who were blinded to genotypes. Images with a total score greater than the average +2SD (rounded up) of the control group were considered positive for autoantibodies.

### Histology

Tissue harvested from WT and CypKO mice were fixed with 4% PFA and sent to the McGill Histology Core Facility for formalin-fixed paraffin-embedded processing, hematoxylin and eosin (H&E) staining, and imaging.

### Gene expression

Up to 5 × 10^6^ cells were lysed, and RNA was extracted using a FavorPrep Total RNA Mini Kit (Favorgen) and quantified with a NanoDrop 2000c (Thermo Fisher Scientific). RNA was converted to cDNA using an AdvanTech 5X master mix. Oligo sequences (available upon request) were designed with Primer3, and primer specificity was verified by BLAST. cDNA (10 ng) was used per qPCR reaction and BlasTaq 2X qPCR MasterMix (abm). qPCR reactions and melting curves were done with a LightCycler 96 (Roche). Gene expression was normalized to 18*S* values or, in some cases, the epithelial marker *Epcam*.

### Preparation of single-cell suspensions

Thymi were cut into 1-mm pieces and digested in freshly prepared RPMI 1640 (Wisent) containing 10% FBS (Millipore Sigma) (R10) and collagenase D (250 μg/ml), papain (250 μg/ml), and deoxyribonuclease I (200 μg/ml) for 30 min at 37°C with shaking (100 rpm). Cells were collected by pipetting up and down. Spleens were crushed through 70-μm filters with the back of a syringe and washed with 10 ml of RPMI + 1% fetal calf serum. After centrifugation, red blood cells were lysed with an ACK lysing buffer (Gibco) for 3 min at RT, washed, and counted.

### Single-cell RNA-seq

Two 8-week-old male *Cyp27b1*^+/+^ and *Cyp27b1*^−/−^ littermates (F2) were euthanized, and TECs were enriched and sorted as described above. Libraries were generated (Génome Québec) using the Chromium Next GEM Single Cell 3′ GEM, Library & Gel Bead Kit v3.1, Chromium Next GEM Chip G Single Cell Kit, and Single Index Kit T Set A (10X Genomics) as per the manufacturer’s recommendations. The targeted cell recovery was set at 3000. Libraries were quantified using the KAPA Library Quantification Kits–Complete kit (Universal) (Kapa Biosystems). The average fragment size was determined using a LabChip GXII (PerkinElmer) instrument. The libraries were normalized and pooled and then denatured in 0.02 N NaOH and neutralized using an HT1 buffer. The pool was loaded at 375 pM on an Illumina NovaSeq SP lane using the Xp protocol as per the manufacturer’s recommendations. The run was performed for PE 28x91. A phiX library was used as a control and mixed with libraries at 1% level. Base calling was performed with RTA v3. bcl2fastq2 v2.20 was then used to demultiplex samples and generate Fastq reads. Cells were analyzed using Seurat. First, filtering for the number of genes, number of unique unique molecular identifiers (UMIs), and mitochondrial content was performed for quality control. Second, all four samples were integrated. The new integrated object was scaled and normalized, and dimensionality reduction was conducted with principal components analysis (PCA) using the first 20 principal components. Last, UMAP plots were constructed, and clusters were identified using the shared nearest neighbor algorithm based on the first 20 principal components. For RNA velocity analysis, loom files were generated for individual samples. RNA velocity was performed with jupyter notebook using scVelo and scanpy libraries. Velocity plots were generated using the dynamical model. A list of 300 DEGs derived from representative CypKO and WT samples were used for IPA.

### Smart-Seq2 realignment and preprocessing

All sequencing data were downloaded from ENA (ENA no. ERP119415). FASTQ files were uploaded to Terra (https://app.terra.bio) and raw sequencing data were mapped and quantified using STAR through the Cumulus workflow (https://cumulus.readthedocs.io/en/stable/smart_seq_2.html; version 1.5.0, default parameters, using GRCm38) to generate raw count, gene-by-cell expression matrices. Aligned matrices were filtered to remove low-quality barcodes [keeping those with >100 genes, <20% mitochondrial reads, and <20% ribosomal reads and a complexity score (log_10_GenesPerUMI) greater than 0.3]. Filtered gene-by-cell matrices were merged and processed using a standard unsupervised workflow using Seurat and scCustomize in R. The merged dataset was normalized and log-transformed (scaling factor = 10,000), and the top 2000 highly variable genes were identified (gene selection method = vst) and used for scaling the data. Dimensionality reduction was then performed by PCA over the top 2000 variable genes, and after exploring individual principal components, the top 40 principal components were selected for visualization. Nearest neighbor graphs and UMAPs were constructed using these top 40 principal components. Cells were clustered using Louvain clustering, and the package clustree was used to generate a clustering tree and identify which resolution achieved stability. Clusters were annotated based on differential gene expression. Heatmaps were generated using the pheatmap package in R.

### Microarray dataset analysis

Differential gene expression data of mTEC^lo^ (GSE56928) (*48*) or total TEC (GSE132278) (*90*) samples relative to 4-week-old samples were generated with GEO2R using pooled replicates when available. Heatmaps were constructed in R using the pheatmap package.

### Glucose tolerance test

Mice were fasted overnight and given glucose (2 mg/kg) diluted in PBS by intraperitoneal injection. Blood was collected by tail vein bleed 0, 15, 30, 60, and 120 min after injection, and glucose was measured with a glucometer (OneTouch Ultra 2).

### Statistical analyses

Statistics were calculated using GraphPad Prism version 8 with parametric unpaired *t* tests. Statistical significance is indicated by *P* values: *P < 0.05; **P < 0.01; ***P < 0.001; ****P < 0.0001.
