# Supplementary material for: Skewed epithelial cell differentiation and premature aging of the thymus in the absence of vitamin D signaling
Source: Sci Adv. 2024 Sep 25;10(39):eadm9582. doi: 10.1126/sciadv.adm9582 (PMC11423877; doi:10.1126/sciadv.adm9582)
Supplement: Supplementary file 1 — Figs. S1 to S11 Table S1 [file sciadv.adm9582_sm.pdf]

Science Advances
AAAS

Supplementary Materials for

Skewed epithelial cell differentiation and premature aging of the thymus in the absence of vitamin D signaling

Patricio Artusa *et al.*

Corresponding author: John H. White, john.white@mcgill.ca

Sci. Adv. **10**, eadm9582 (2024)
DOI: 10.1126/sciadv.adm9582

This PDF file includes:

Figs. S1 to S11
Table S1

Supplementary Materials:

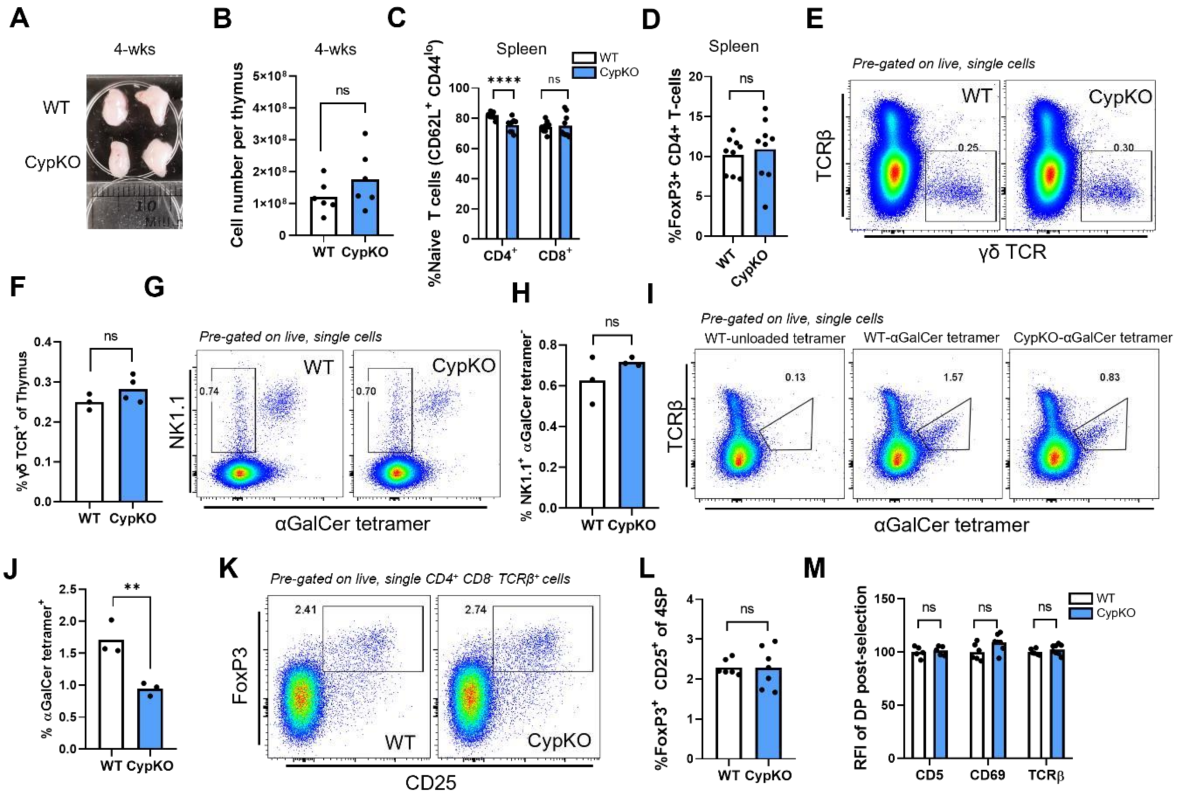

fig S1. Altered T cell development and conventional T cell homeostasis in CypKO mice A. *Thymic lobes from 2 WT (top) and KO (bottom) 4-week-old mice sex-matched mice.* ***B.*** *Total thymic cell number in 4-week-old WT versus KO mice.* ***C.*** *Frequency of naïve (CD62L$^+$ CD44^{lo}) splenic CD4$^+$ and CD8$^+$ T cells.* ***D.*** *Frequency of splenic FoxP3$^+$ regulatory T cells.* ***E,F.*** *Representative flow cytometry plots of γδ T-cells in WT vs KO mice (E) and summary data (F).* ***G,H.*** *Representative flow cytometry plots of NK cells in WT vs KO mice (G) and summary data (H).* ***I,H.*** *Representative flow cytometry plots of αGalCer tetramer$^+$ iNKT cells in WT vs KO mice (G) and summary data (H).* ***K,L.*** *Representative flow cytometry plots of regulatory T cells in WT vs KO thymi (K) and summary data (L).* ***M.*** *Relative fluorescence intensity (RFI) of TCR signaling molecules on post-selection DP thymocytes, relative to average WT expression. All experiments were performed with age-matched (male and female) 6-10-week old mice. Each dot represents an individual mouse. Statistics: Unpaired parametric T tests. p<0.05*, p<0.01**, p<0.001***, p<0.0001****.*

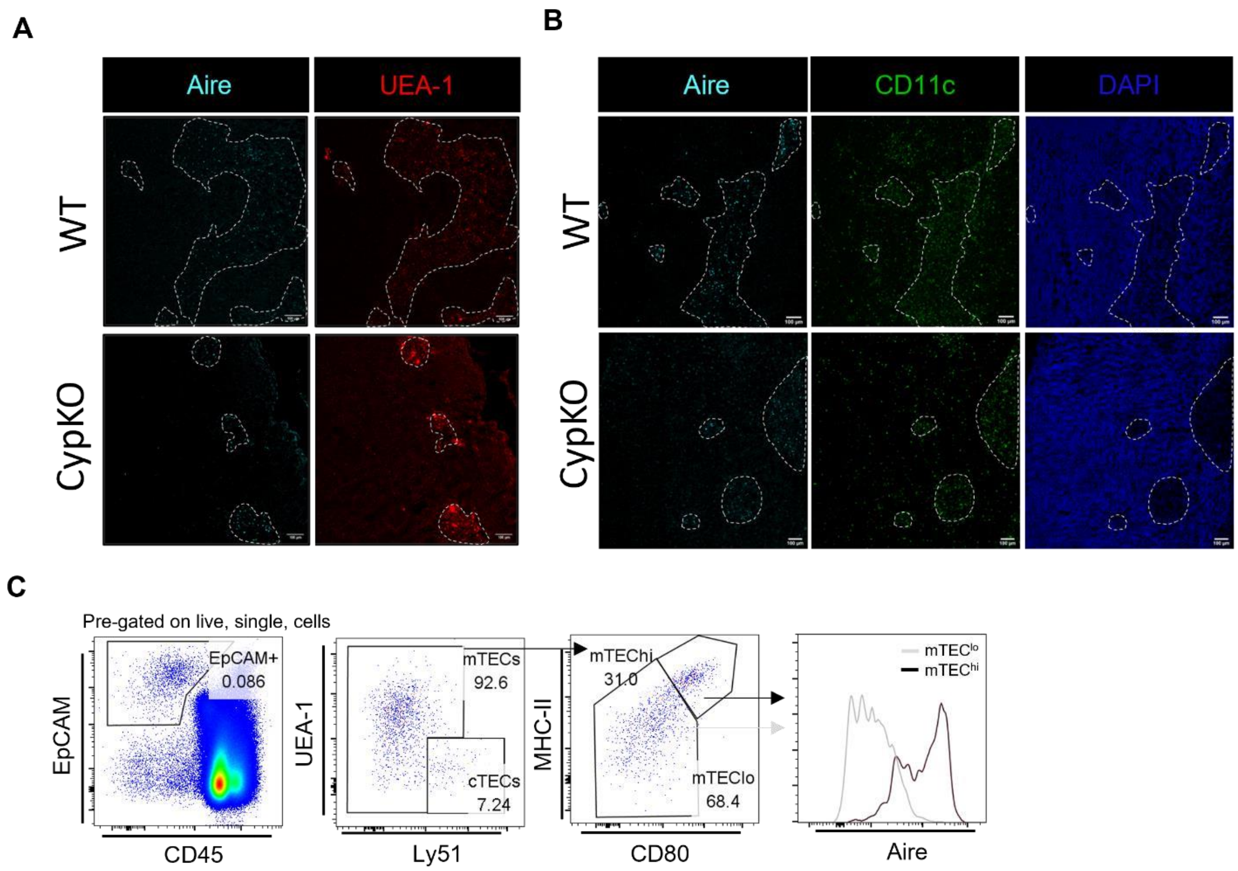

fig S2. Defined medullary structures in CypKO thymi. A. *Representative IF microscopy images of Aire (in white dashed lines) and the mTEC marker, UEA-1, staining in WT and CypKO thymi.* ***B.*** *Representative IF microscopy images of Aire, CD11c, and DAPI with distinctive medullary (Aire^{+} regions, white dashed lines) staining patterns in both WT and CypKO thymi.* ***C.*** *Flow cytometric-gating strategy for TEC populations. Experiments were performed with age-matched (male and female) 8-10-week old mice.*

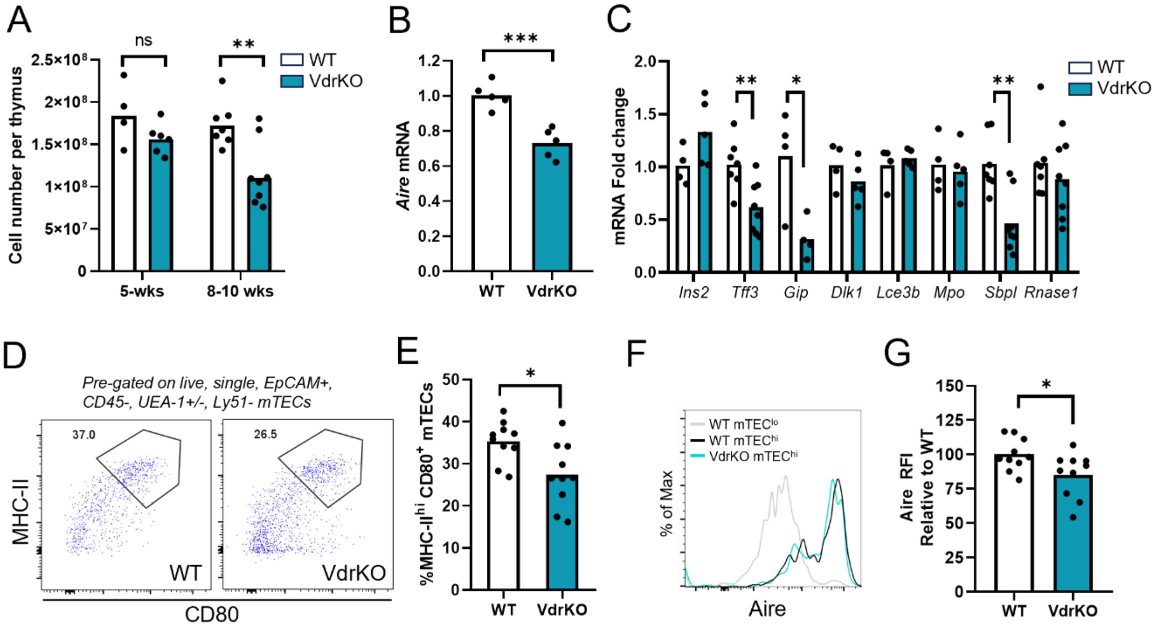

fig. S3. Impaired Aire expression and thymic development in VdrKO mice. A. *Total thymic cell number in 5- or 8-9 week old WT and VdrKO littermates.* ***B,C.*** *Aire (B) and TRA (C) gene expression in WT versus VdrKO thymic samples.* ***D.E.*** *Representative flow cytometric gating of mTEC^{hi} cells (MHC-II^{hi} CD80^{+}) in WT and VdrKO thymi (D) and quantification of their frequencies (E) from three independent experiments.* ***F,G.*** *Representative flow cytometry plot of Aire expression in WT and VdrKO mTEC^{hi} cells (F), mTEC^{lo} as staining control, and quantification from two independent experiments (G). Sex-matched (male and female) mice were used for figure A. Age- and sex-matched 8-10-week-old mice were used for figures B-G. Each dot represents an individual mouse. Statistics: Unpaired parametric T tests. p<0.05*, p<0.01**, p<0.001***, p<0.0001****.*

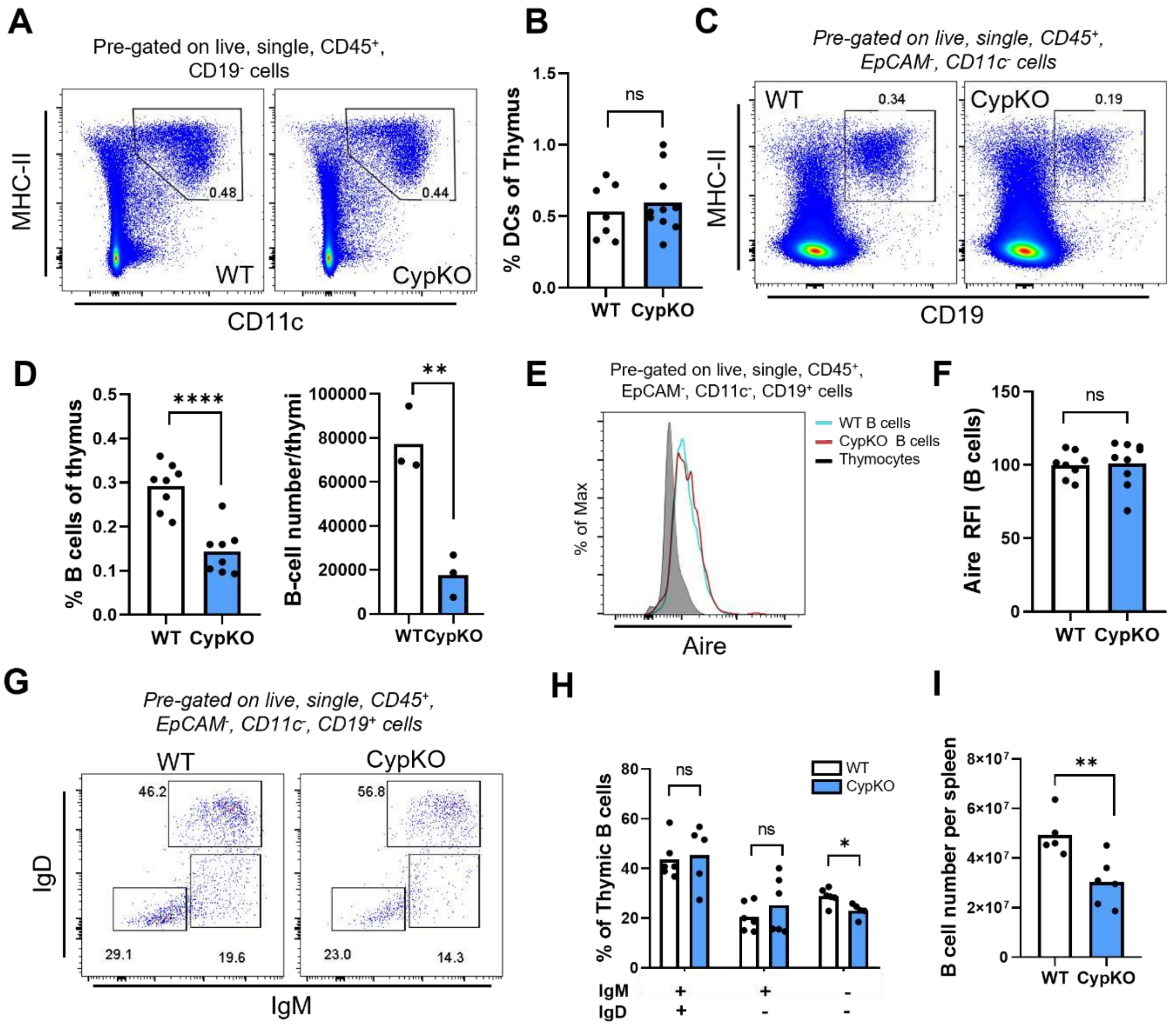

fig S4. Systemic B cell lymphopenia in CypKO mice A,B. *Representative flow cytometry plots of dendritic cells (DCs) in WT vs KO mice (A) and summary data (B).* ***C,D.*** *Representative flow cytometry plots of B cells in WT and KO thymi (C) and summary data (D).* ***E,F.*** *Representative flow cytometry plot of Aire expression in thymic B cells (E) and quantification of the relative fluorescence intensity (relative to average of WT expression) (F).* ***G.*** *Representative flow cytometry plots of thymic B cell subsets (IgD^{+} IgM^{+}; "recirculating", IgM^{+} IgD^{-}/IgM^{-} IgD^{-}; "resident").* ***H.*** *Quantification of committed and "circulating" B cell subtypes in WT versus KO thymi.* ***I.*** *Splenic B cell (Live, single, CD19^{+} MHC-II^{+}) frequencies. All experiments were performed with age-matched (male and female) 6-10-week old mice. Each dot represents an individual mouse. Statistics: Unpaired parametric T tests. p<0.05*, p<0.01**, p<0.001***, p<0.0001****.*

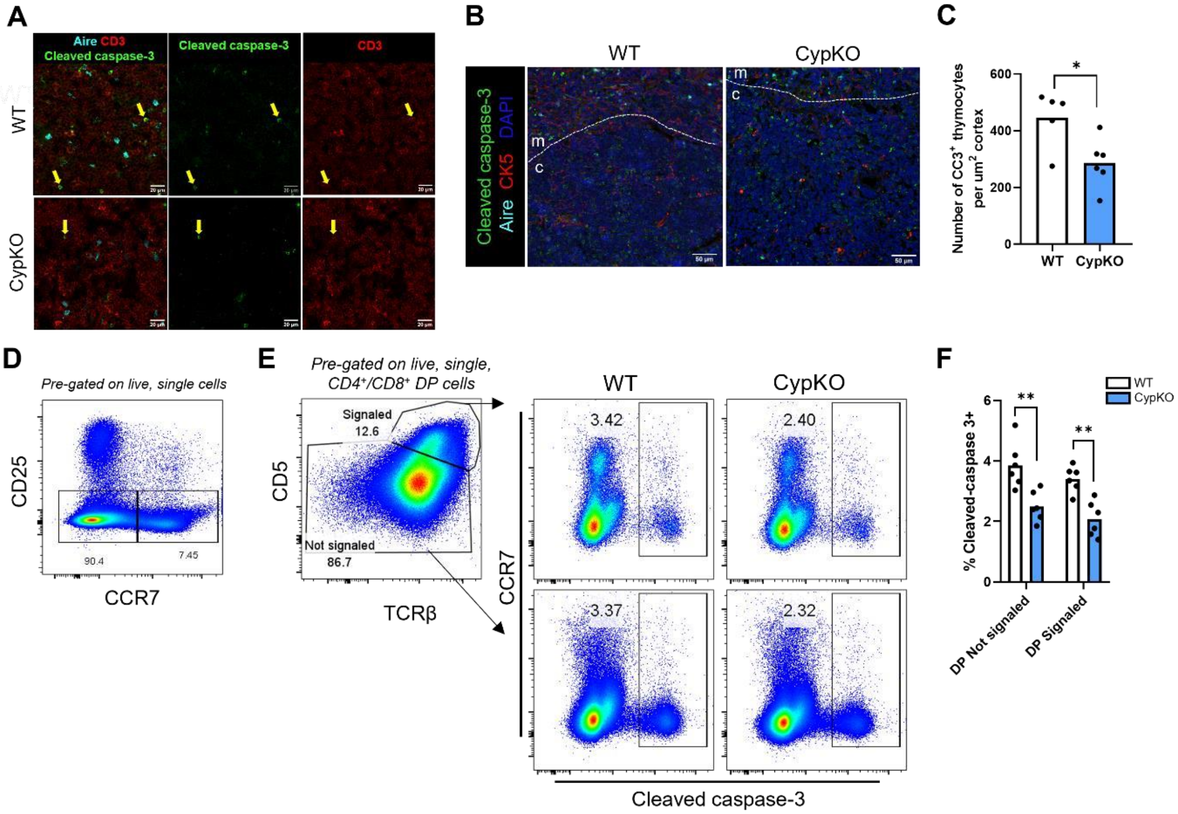

fig S5. Decreased thymocyte apoptosis in Cyp27b1 KO mice A. *Thymic IF microscopy images showing co-staining of cleaved caspase 3 (small rings) with CD3^{+} thymocytes.* ***B,C.*** *Representative IF microscopy images showing cleaved caspase 3 staining in the thymic cortex (Aire^{-} regions)(B), and representative summary data (C) (c=cortex, m=medulla).* ***D.*** *Flow cytometry plot showing gating for CCR7^{+} and CCR7^{-} CD25^{-} populations.* ***E.*** *Flow cytometric gating strategy for "signaled" (CD5^{+} TCRβ^{hi}) and "not signaled" (CD5^{-}, TCRβ^{lo}) DP thymocytes* ***F.*** *Summary of the frequency of cleaved caspase 3+ "signaled" and "not signaled" DP thymocytes in WT vs Cyp27b1 KO thymi. All experiments were performed with age-matched (male and female) 8-12-week old mice. Each dot represents an individual mouse. Statistics: Unpaired parametric T tests. p<0.05*, p<0.01**, p<0.001***, p<0.0001****.*

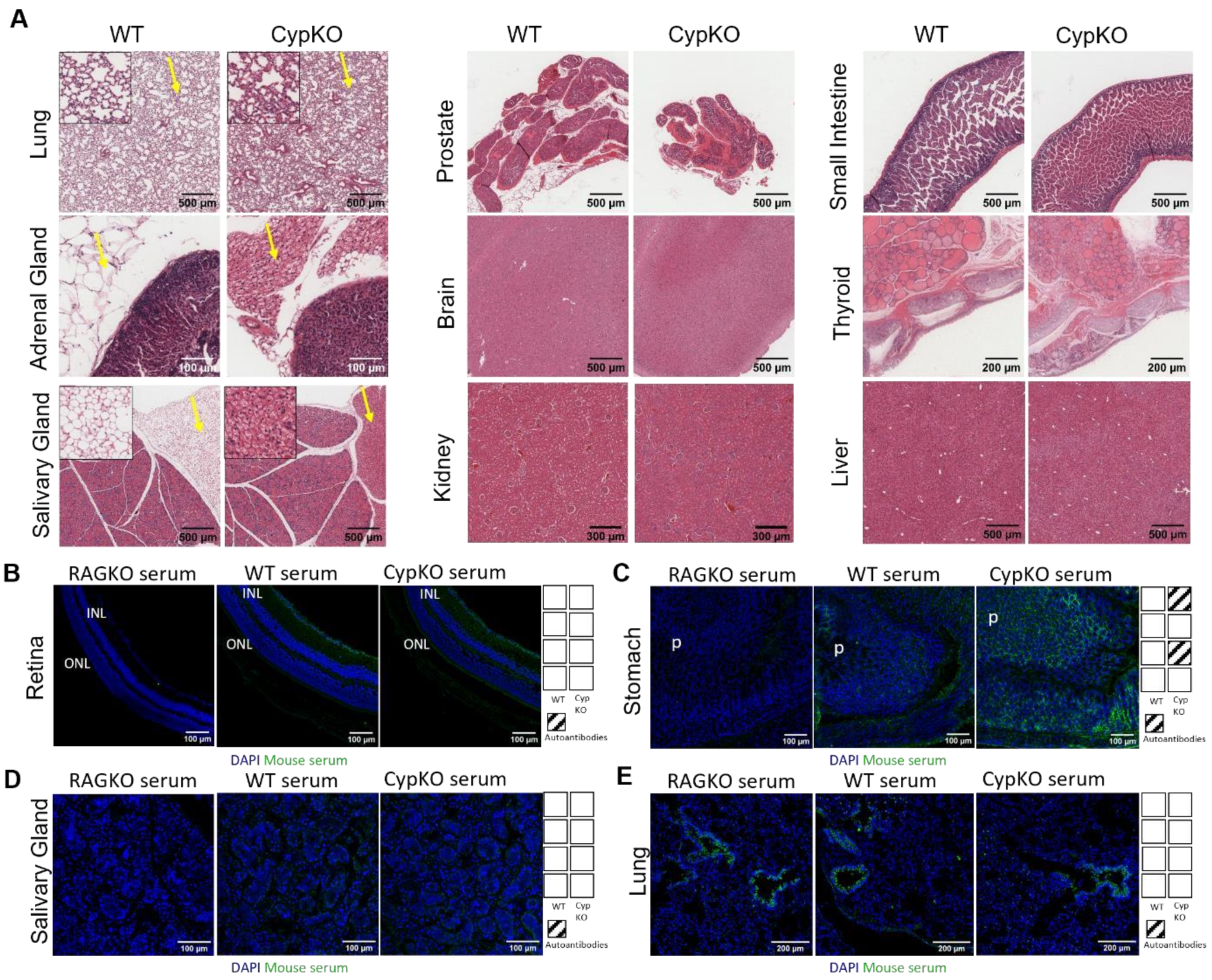

fig. S6. Mild inflammation in several CypKO mouse tissues. A. *Representative H&E images of tissue from 26-week-old WT or CypKO mice (male).* ***B-E***. *Representative confocal microscopy images of autoantibody staining of RagKO, WT, or CypKO serum samples on the indicated target organs and summary of phenotypic penetrance. Experiments were performed with male 26-week-old mice.*

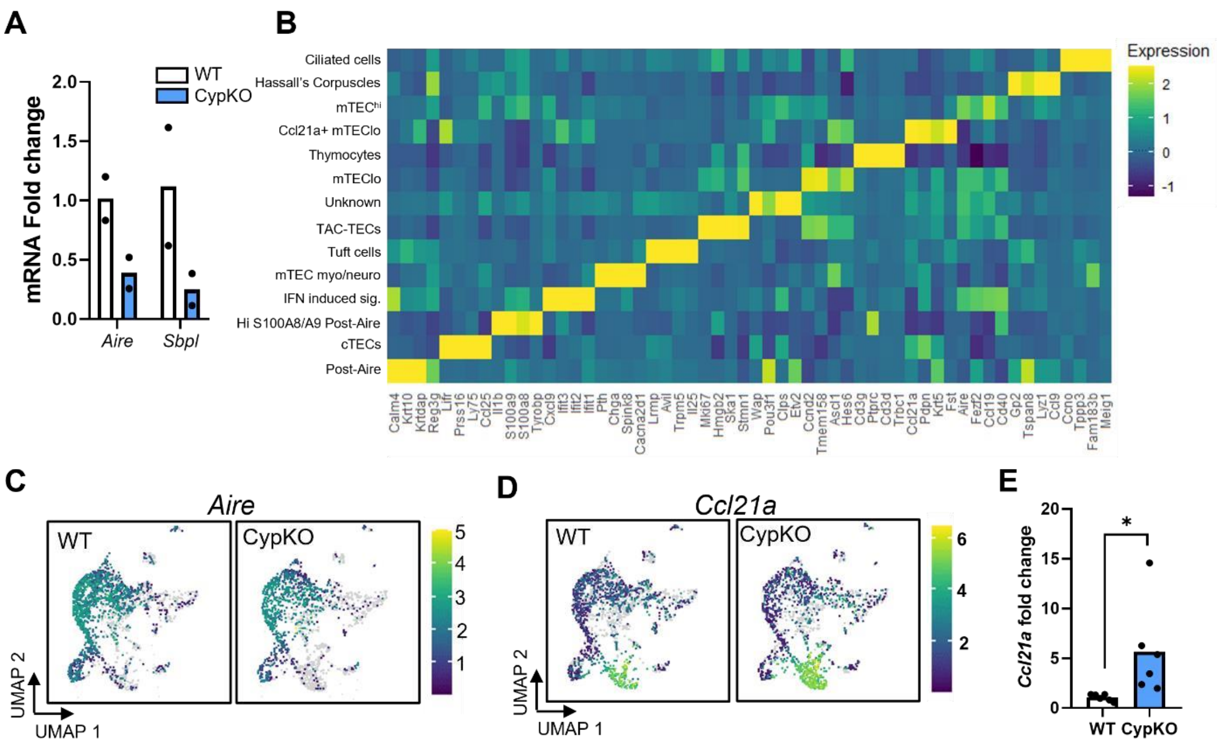

fig S7. Divergent mTEC differentiation in CypKO mice A. *qPCR data from sequenced samples.* ***B.*** *Heatmap of 4 top 20 DEGs per cluster.* ***C.*** *UMAPs showing Aire expression in WT and KO samples.* ***D.*** *UMAPs showing Ccl21a expression in representative WT versus KO samples.* ***E.*** *qPCR validation of Ccl21a expression**.** Experiments in (E) were performed with age-matched (male and female) 8-12-week old mice. Each dot represents an individual mouse. Statistics: Unpaired parametric T tests. p<0.05*, p<0.01**, p<0.001***, p<0.0001****.*

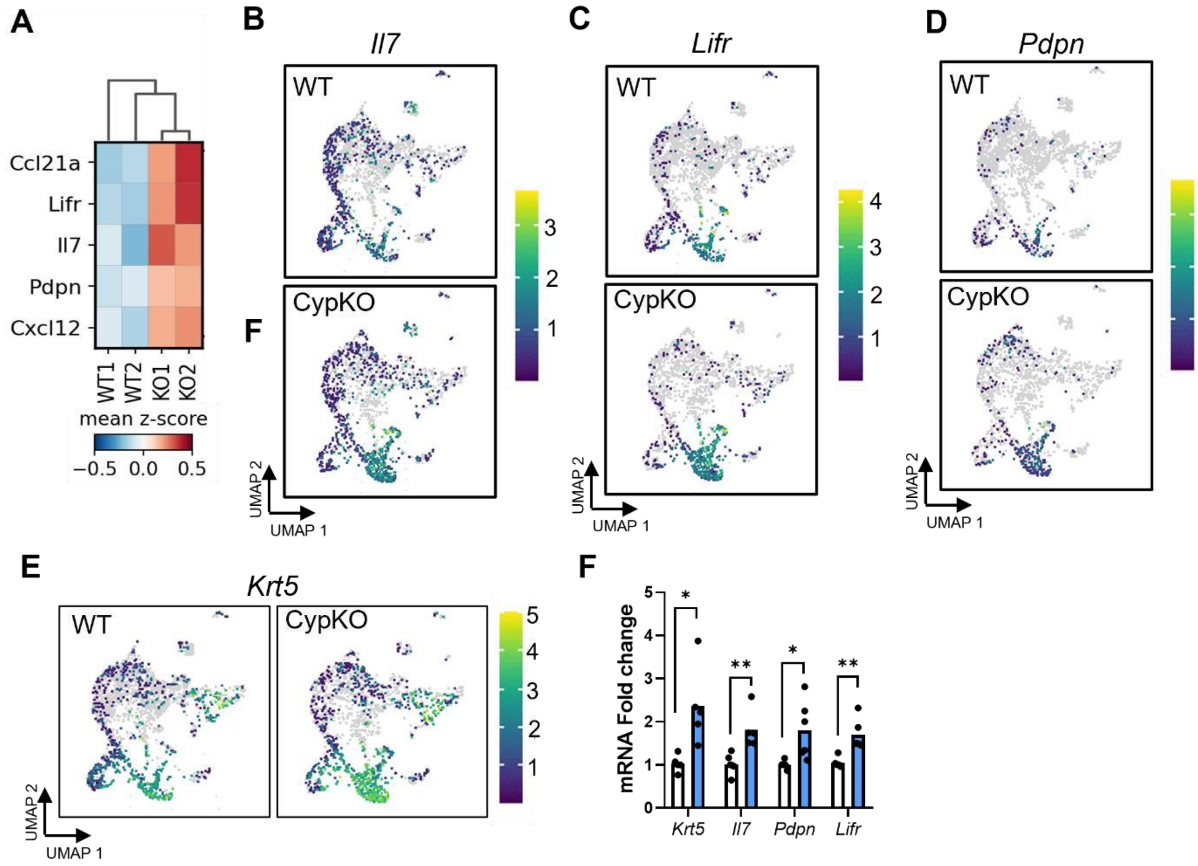

fig S8. Increased "intertypical" TEC marker gene expression in CypKO mice A. *Matrixplot of jTEC genes in all sequenced samples.* ***B-D*** *UMAPs showing decreased expression of "intertypical" TEC markers, Il7, Lifr, Pdpn, in the Ccl21a+ cluster.* ***E.*** *UMAP showing increased Krt5 transcripts in KO Ccl21a^{+} TECs.* ***F.*** *RT/qPCR validation of "intertypical" TEC marker genes. Experiments in (F) were performed with age-matched 8-12-week old (male and female) mice. Each dot represents an individual mouse. Statistics: Unpaired parametric T tests. $p<0.05$*, $p<0.01$**, $p<0.001$***, $p<0.0001$****.*

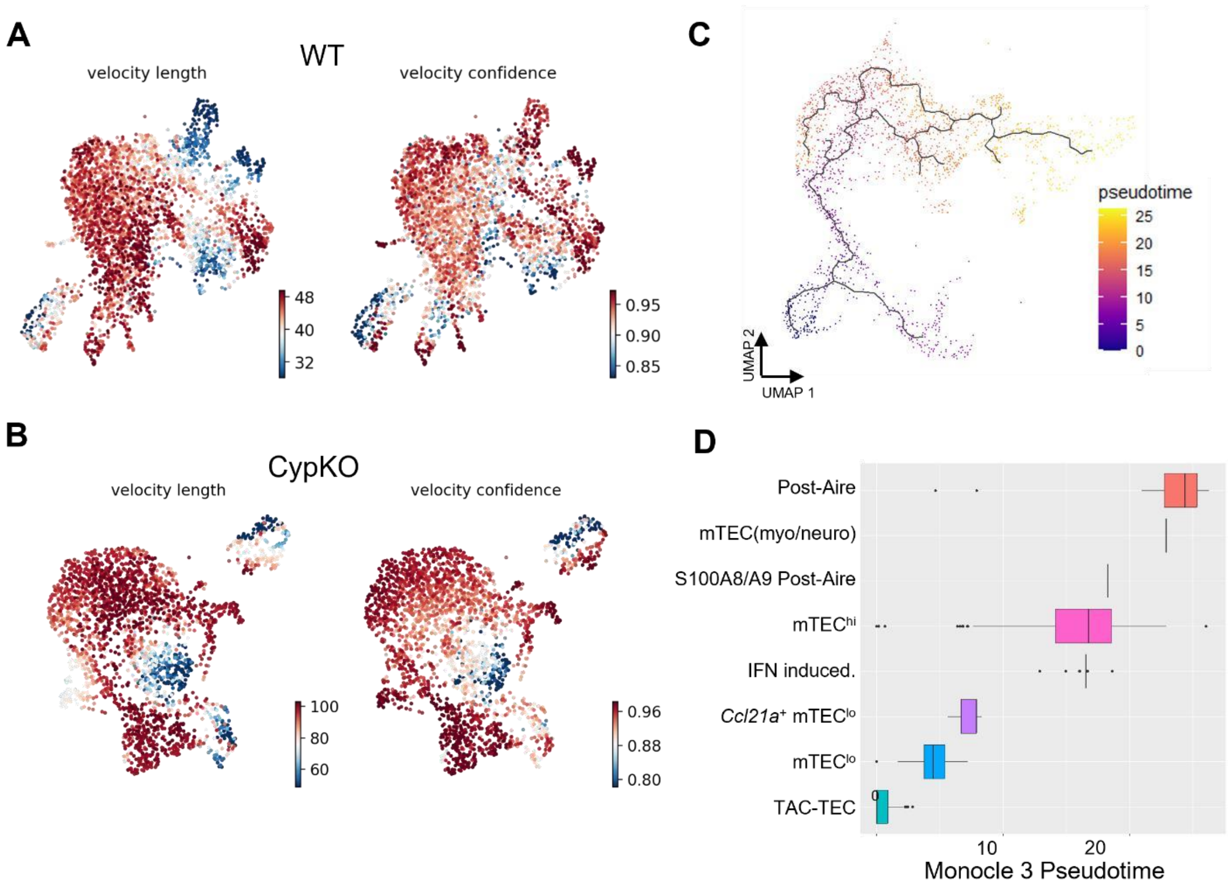

fig S9. Trajectory analysis A. *Velocity length (left) and velocity confidence (right) for WT.* ***B.*** *Velocity length (left) and velocity confidence (right) for CypKO.* ***C.*** *Pseudotime projection of a representative WT TEC sample.* ***D.*** *Pseudotemporal ordering of TEC clusters in a WT sample***.**

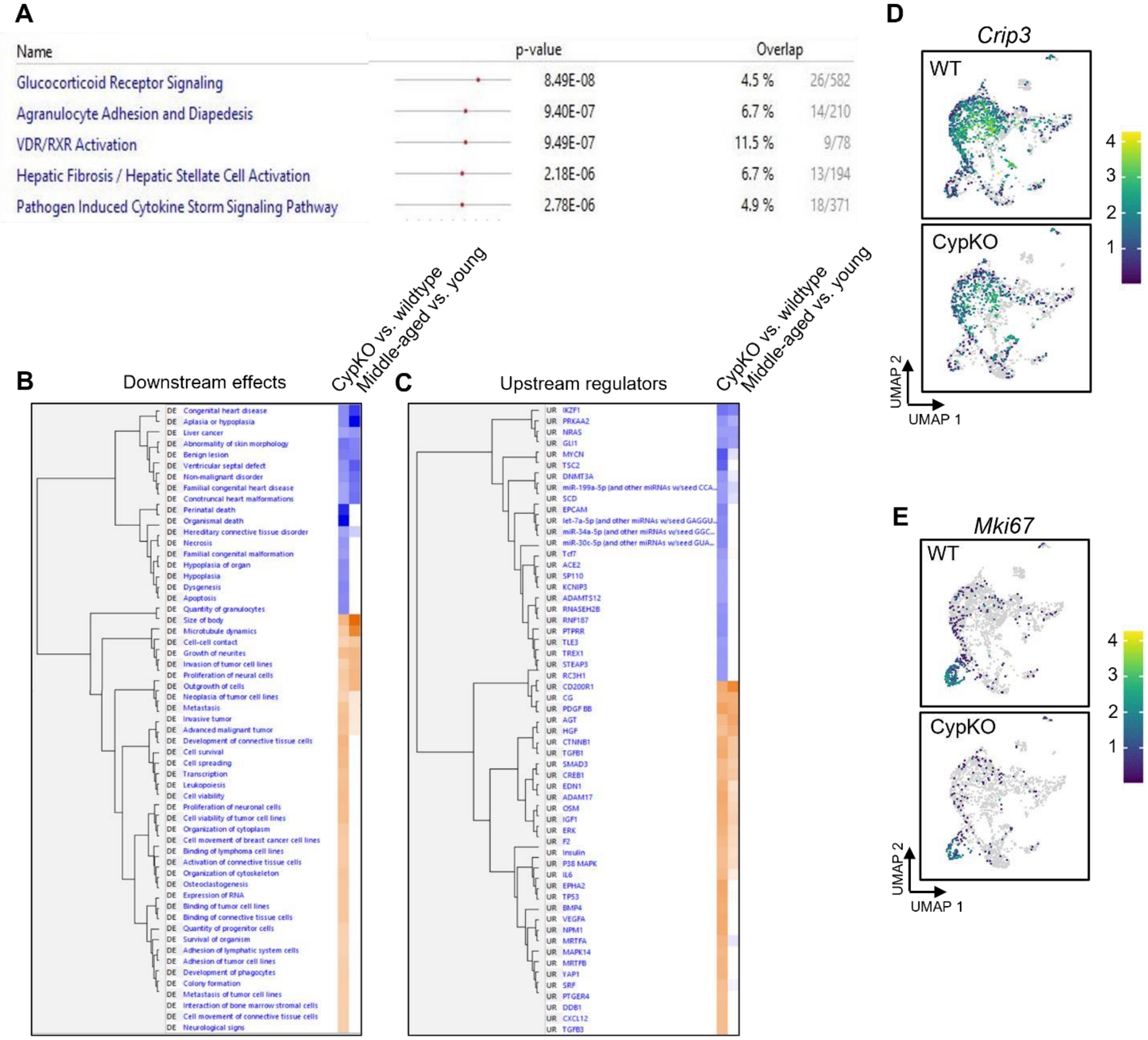

fig S10. Enrichment of aging pathways in CypKO A. *Top canonical pathways in Cyp27b1 KO vs WT TECs.* ***B.*** *Comparison of downstream effects (Z-score 45.83%) between our dataset and a middle-aged mouse versus young mouse dataset.* ***C.*** *Comparison of upstream regulators (Z-score 51.30%) between our dataset and a middle-aged mouse versus young mouse dataset.* ***D.*** *Representative UMAPs of Crip3 expression in WT and KO TECs.* ***E.*** *Representative UMAPs of Mki67 expression in wildtype and KO TECs.*

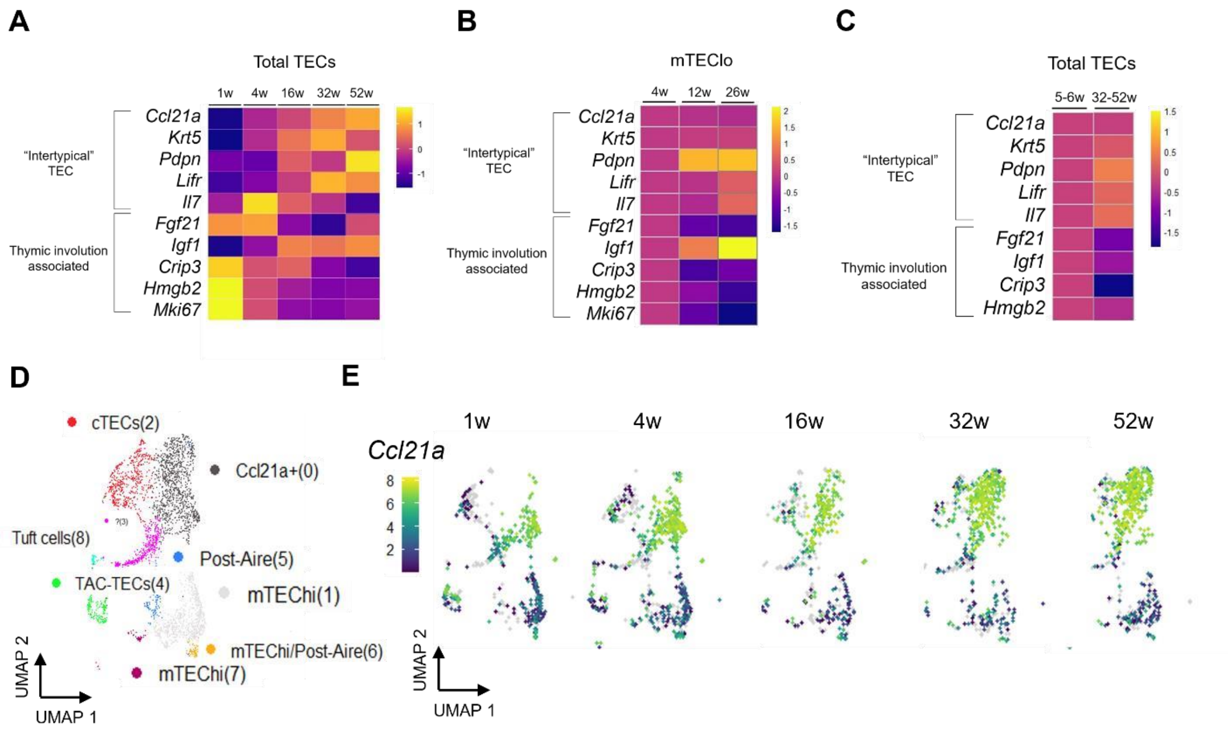

fig S11. Enhanced "intertypical" TEC gene signature in aging thymi. ***A.*** *Average expression of "Intertypical" TEC and thymic involution-associated genes in TECs from 1-52-week-old mice. (Raw scRNA-seq data from Garan-Bale et al. eLife 2020 9:e56221)* ***B.*** *Fold change of "Intertypical" TEC and thymic involution associated genes in mTEC^{lo} cells from 4-26-week-old mice. (Raw data by microarray from Ki S. et al. Cell Rep. 2014 Oct 9;9(1):402-415.)* ***C.*** *Fold change of "Intertypical" TEC and thymic involution-associated genes in TECs from 5-52-week-old mice. (Raw data by microarray from Lepletier A. et al. Cell Rep 2019 Jun 25;27(13):38873901.e4.)* ***D.*** *Cluster annotation of integrated scRNA-seq data from 1-52-week-old mouse TECs (Raw data from Garan-Bale et al. eLife 2020 9:e56221)* ***E.*** *UMAPs showing Ccl21a gene expression in TECs from 1–52-week-old mice. (Raw data from Garan-Bale et al. eLife 2020 9:e56221.)*

table S1. List of differentially expressed genes by cluster.

mTEC^{hi}		
**Gene Name**	**avg_log2FC**	**p_val_adj**
Mt4	2.248	4.94E-213
S100a8	2.080	8.96E-203
Crhbp	1.997	0
Fabp6	1.969	1.11E-79
Lypd8	1.942	2.81E-57
Pcp4	1.835	7.35E-61
Obp2a	1.661	1.11E-205
Sncg	1.648	4.52E-176
Pyy	1.635	1.03E-73
Defb19	1.631	2.60E-101
S100a9	1.559	1.49E-82
Crip1	1.557	2.46E-135
Tbc1d4	1.557	0
S100a6	1.556	2.73E-137
AW112010	1.552	3.30E-121
Ccl27a	1.547	3.96E-195
Calca	1.542	3.33E-97
Fgf21	1.528	3.07E-203
Pomc	1.437	5.98E-107
Nts	1.434	6.41E-15
Crabp1	1.404	3.62E-28
Csn2	1.397	2.41E-239
Lgals2	1.389	1.18E-67
Calca	1.369	7.16E-110
Gal	1.369	0.015902959

Ccl21a^{+}, mTEC^{lo}		
**Gene Name**	**avg_log2FC**	**p_val_adj**
Ccl21a	4.253	0
Apoe	2.998	0
Ifi27l2a	2.734	0
Krt5	2.688	0
Tagln	2.645	4.11E-250
Ccl11	2.629	6.02E-158
Mgp	2.473	0
Krt14	2.450	1.67E-303
Ctsl	2.325	0
Acta2	2.224	5.01E-105
Igfbp5	2.201	3.83E-230
Lifr	1.984	0
Socs3	1.978	1.09E-243
Id1	1.930	0
Gas1	1.898	0
Hpgd	1.864	1.96E-287
Rbp1	1.681	9.30E-303
Igfbp4	1.640	9.49E-235
Ascl1	1.639	4.29E-216
Ly6a	1.488	8.29E-159
Isg15	1.485	1.30E-182
Gpx3	1.477	4.27E-136
Atf3	1.471	2.38E-85
Fst	1.433	5.93E-254
Ifit3	1.407	1.99E-165

?		
**Gene Name**	**avg_log2FC**	**p_val_adj**
Gm26917	2.48199085	9.91E-16
Wap	0.69027834	0.15482836
Lifr	0.66283792	1.24E-57
Fgl2	0.50693686	1
Snhg11	0.48815608	0.28274831
Mt2	0.48398488	1
Clps	0.48309626	1
Ccl8	0.4821778	1.89E-13
Cbr2	0.4261964	1.44E-08
Krt5	0.42305376	3.32E-08
Gm8113	0.3991534	0.03279093
Tchh	0.36997676	4.50E-16

Post-Aire		
**Gene Name**	**avg_log2FC**	**p_val_adj**
Calm4	4.487	1.22E-176
Krtdap	4.485	1.46E-201
Krt10	4.074	3.36E-209
Reg3g	3.955	1.68E-23
Sbsn	3.940	1.27E-222
Wfdc2	3.927	6.95E-70
Dapl1	3.914	9.90E-239
Lgals7	3.775	5.78E-160
Krt16	3.773	2.39E-157
Crct1	3.506	2.83E-200
Dmkn	3.452	1.79E-212
Calml3	3.342	1.01E-197

Etv2	0.34005012	5.85E-22
Pou3f1	0.33716992	1.45E-30
Ccl25	0.33624516	0.00364013
Rgs4	0.33450063	3.64E-10
Jchain	0.33320809	2.58E-34
Pds5b	0.32924118	0.11136192
Defb3	0.31870468	0.00181596
Igfbp5	0.31811168	1.29E-05
Ceacam10	0.31277888	3.63E-09
Cd44	0.29337331	1.33E-17
Fn1	0.29183855	8.00E-21
F830016B08Rik	0.2889918	1
Ccl21a	0.27555925	2.22E-16

Spink5	3.233	5.46E-155
Defb6	3.198	4.21E-140
Krt79	3.161	3.64E-173
Scgb1a1	3.147	6.02E-19
Anxa1	3.143	1.66E-64
Ifitm1	3.070	2.27E-44
Ltf	3.038	3.71E-59
Spink1	3.025	4.64E-10
Gsto1	2.971	1.45E-36
Ly6g6c	2.854	8.40E-157
Cnfn	2.841	5.24E-182
Gm94	2.805	2.29E-105
Spink5	2.796	7.91E-54

mTEC^{lo}

Gene Name	avg_log2FC	p_val_adj
Ccnd2	2.033	2.16E-173
Tmem158	1.648	4.32E-116
Ascl1	1.604	2.24E-144
Hes6	1.513	2.73E-142
Stmn1	1.237	1.71E-117
Tubb5	1.135	2.01E-122
Dut	1.074	1.81E-121
Nkx6-2	1.065	1.00E-99
Fgf21	0.955	8.44E-78
Vim	0.924	6.37E-95
H2afz	0.901	6.18E-67
Hmgb2	0.853	1.41E-29
Olfm4	0.771	3.90E-63
Hist1h1b	0.763	3.51E-31
Pclaf	0.739	1.94E-72
Ttn	0.716	1.02E-58
Hells	0.677	2.25E-30
Hsp90aa1	0.665	1.93E-67
Ltb	0.652	2.48E-76
Cks1b	0.651	5.34E-99
Wfdc18	0.635	5.70E-60
Tuba1b	0.587	1.86E-43
Rgs5	0.564	5.33E-56
Tyms	0.528	1.01E-50
Uhrf1	0.510	1.28E-51

Tuft cells

Gene Name	avg_log2FC	p_val_adj
Lrmp	3.668	4.93E-123
Gnat3	3.039	1.57E-56
Plac8	2.942	9.21E-71
Rgs13	2.937	6.22E-27
Gng13	2.891	5.78E-66
Avil	2.735	1.54E-78
Plk2	2.538	8.37E-70
Rgs2	2.522	1.46E-62
Mctp1	2.498	2.20E-65
Alox5ap	2.488	4.27E-43
Gnb3	2.435	2.21E-21
Fyb	2.333	2.13E-37
Atp1a2	2.318	1.58E-70
Trpm5	2.248	2.90E-44
Atp2a3	2.239	2.04E-56
Aldh2	2.189	9.67E-41
Ptpn18	2.184	6.79E-40
Bmx	2.126	4.98E-20
Dclk1	2.123	9.47E-10
Ahnak2	2.119	5.12E-53
Ltc4s	2.097	6.54E-64
Plcb2	2.041	3.06E-39
Ly6g6f	1.981	6.86E-97
Cd24a	1.924	2.12E-41
Cited2	1.919	3.33E-39

TAC-TECs

Gene Name	avg_log2FC	p_val_adj

Hassall's corpuscles

Gene Name	avg_log2FC	p_val_adj

Hmgb2	2.995	3.97E-200	Serpinb6a	5.027104	1.28E-111
Stmn1	2.586	3.65E-186	Ccl20	4.753318	1.03E-81
Hist1h1b	2.296	4.37E-134	Pglyrp1	4.599647	2.99E-71
Pclaf	2.252	5.95E-189	Lyz1	4.331567	6.32E-54
Tubb5	2.160	7.12E-173	Ccl9	4.249893	2.18E-122
Mki67	2.074	5.48E-189	Ccl6	4.176278	9.17E-89
Ube2c	2.031	1.54E-148	Wfdc17	3.974778	1.04E-70
H2afz	1.981	1.57E-180	Serpinb1a	3.879944	2.26E-110
Cdk1	1.966	1.47E-192	Wfdc18	3.795423	7.01E-54
Top2a	1.958	2.21E-152	Hamp	3.647744	1.69E-53
Cks2	1.897	7.40E-171	Gp2	3.269319	4.05E-55
Dut	1.837	1.07E-162	2200002D01Rik	3.191545	2.55E-106
Birc5	1.758	4.36E-199	Iapp	3.100513	3.57E-40
Tuba1b	1.744	1.22E-140	AW112010	3.014389	3.94E-84
Cks1b	1.728	1.54E-185	Cyp2a5	2.934383	4.58E-66
Hist1h2ae	1.632	3.60E-133	Tnfaip2	2.900161	4.60E-44
Cenpf	1.579	1.39E-138	Clu	2.876343	4.81E-56
Hist1h2ap	1.523	7.11E-75	Bcl2a1b	2.841082	7.48E-94
Tpx2	1.512	5.12E-148	Prg2	2.831532	1.13E-11
Cdca8	1.490	2.18E-186	H2-M2	2.793833	1.02E-57
H2afx	1.474	4.71E-153	Mmp7	2.785579	1.65E-64
Tyms	1.447	1.33E-159	Fxyd2	2.657434	2.48E-45
Hmmr	1.417	4.66E-109	Bcl2a1a	2.623822	1.26E-76
Cdc20	1.412	3.63E-115	Tnfrsf11b	2.572307	2.43E-61
Nusap1	1.339	1.07E-164	Reg3g	2.472904	9.93E-11

Thymocytes

Gene Name	avg_log2FC	p_val_adj
Trbc2	5.289937099	2.94E-111
Cd3g	4.09051625	1.28E-114
Trbc1	4.080953087	6.02E-69
Satb1	3.939927275	4.63E-92
Cd3d	3.821076309	1.01E-111
Itm2a	3.617645267	5.05E-73
Ptprc	3.563212201	1.20E-112
Lef1	3.174798469	4.47E-66
Rgs10	2.399626311	2.03E-51
Ptpn18	2.276409979	7.53E-88
Dusp5	2.012075433	3.16E-69
Ccr7	1.92264816	3.69E-71
Ms4a4b	1.871074859	6.02E-64
Nr4a1	1.796812002	6.81E-86
Hist1h1e	1.682397533	9.53E-29
Ltb	1.637667745	9.72E-27

IFN inducible signature

Gene Name	avg_log2FC	p_val_adj
Cxcl9	2.702234	2.34E-24
Ifit2	2.546196	2.16E-33
Ifit3	2.523433	3.13E-31
Isg15	1.986724	1.00E-26
Cxcl10	1.971796	9.33E-28
Krt71	1.888625	0.000161
Iapp	1.817474	7.41E-38
Ifit1	1.809088	9.12E-35
Igtp	1.725054	3.41E-51
Ppy	1.608623	5.23E-48
Rsad2	1.590612	2.39E-36
Pyy	1.558592	8.62E-46
Serpinb12	1.465139	2.43E-15
Ripply2	1.437128	0.000581
Irgm1	1.409331	1.69E-36
Gbp4	1.37809	9.68E-42

Dusp2	1.635942709	1.41E-35	Psca	1.367316	2.66E-10
Nkg7	1.580126901	3.67E-74	Krt9	1.331516	0.536792
Fyb	1.483633168	2.39E-49	Ifit3b	1.301972	6.70E-39
Klk8	1.44972444	1.06E-46	Iigp1	1.281621	2.15E-31
Izumo1r	1.446498726	7.89E-39	F830016B08Rik	1.269348	1.19E-45
Rgs2	1.316672649	6.16E-17	Gbp2	1.225642	0.395441
Rgs1	1.22188448	2.11E-72	Oasl1	1.159475	1.88E-48
Il2rb	1.135570329	1.79E-75	Ido1	1.117974	2.64E-38
Vim	1.089528504	6.73E-29	Ccl5	1.107096	8.38E-34

cTECs		
**Gene Name**	**avg_log2FC**	**p_val_adj**
Prss16	5.058749	1.00E-82
Ctsl	4.714356	1.10E-76
Ccl25	4.200714	1.02E-74
Cxcl12	4.12089	6.84E-82
Tbata	3.811822	4.05E-76
Fxyd2	3.538419	7.38E-64
Psmb11	3.460467	5.64E-57
Ly75	3.217974	9.16E-68
Synm	2.970979	5.12E-60
Isg20	2.630241	4.86E-47
Snhg11	2.612262	1.86E-28
Lifr	2.596734	5.68E-47
Pltp	2.5237	3.42E-58
Hpgd	2.246505	1.92E-42
Cbr2	2.185294	4.64E-40
Cd83	2.031487	1.53E-42
S100a10	1.909181	3.91E-34
Igfbp5	1.83543	5.30E-25
Slc22a7	1.733237	4.48E-39
Inmt	1.567906	0.036766
Mgll	1.530225	2.02E-37
Oat	1.523112	6.61E-31
Oma1	1.445167	1.60E-13
Crip3	1.429452	8.49E-24
Nefm	1.324084	2.12E-42

mTEC myo/neuro (cluster 15, 18		
**Gene Name**	**avg_log2FC**	**p_val_adj**
Pth	8.983393	6.40E-41
Chga	7.102812	1.52E-40
Spink8	3.367312	5.52E-40
Cacna2d1	3.15845	9.37E-52
Rbp4	3.073776	8.74E-27
Cryba2	2.940445	1
Aqp5	2.906813	1.81E-38
Mt3	2.883257	1
Igfbp5	2.601551	4.51E-31
Clca3b	2.601244	5.15E-10
Cd177	2.580144	7.25E-19
Chga	2.488321	1.01E-43
Ascl1	2.471709	3.93E-33
Mafb	2.325713	8.09E-10
Cited2	2.231279	1.15E-28
Scg2	2.09946	5.17E-29
Car8	1.948816	1.07E-13
Bex1	1.940322	7.18E-38
Fbxo2	1.906263	1.17E-34
Tspan1	1.852305	1.10E-37
Fam183b	1.839199	2.04E-21
Ceacam10	1.836163	1.69E-16
Cd34	1.828835	0.238101
Timp2	1.783631	3.30E-20
Btg2	1.747824	1.69E-33

Ciliated cells		
**Gene Name**	**avg_log2FC**	**p_val_adj**
Dynlrb2	3.153538	1.32E-44
Tppp3	3.103299	3.07E-23
Fam183b	3.094136	9.04E-38
Hsp90aa1	3.034659	6.89E-37

Hi S100A8/A9 Post-Aire		
**Gene Name**	**avg_log2FC**	**p_val_adj**
Il1b	4.797871	1.92E-37
S100a9	4.583299	7.82E-05
Tyrobp	4.185418	5.12E-38
Retnlg	3.491873	8.02E-26

Meig1	2.942893	2.93E-35	S100a8	3.219853	0.009796
Mlf1	2.792293	1.02E-41	Cxcl2	3.015568	2.32E-18
4933434E20Rik	2.782433	1.08E-32	Fcer1g	3.00803	1.28E-27
Nudc	2.670647	8.05E-40	Ccl17	2.895047	4.25E-20
Tubb4b	2.639933	8.94E-35	Cst3	2.719647	1.61E-17
Lrrc23	2.621714	1.94E-37	Ccl22	2.704525	3.46E-17
Ccno	2.609096	8.12E-37	Rgs1	2.517134	1.22E-26
Rsph1	2.571079	1.71E-31	G0s2	2.506569	1.33E-21
Tmem107	2.544863	7.47E-31	Lgals3	2.458862	1.10E-29
Tekt1	2.49657	1.39E-40	Lst1	2.452535	1.03E-31
Cks2	2.423038	1.45E-35	Samsn1	2.44638	5.97E-13
Deup1	2.371027	2.46E-31	Ptprc	2.42692	7.40E-39
Vpreb3	2.332935	2.49E-23	Gngt2	2.218463	1.36E-28
Foxj1	2.324588	5.30E-38	Spi1	2.17089	6.77E-24
1110004E09Rik	2.315511	5.42E-40	Ccr7	2.145469	4.85E-14
Ly6c1	2.314051	2.66E-11	Retnla	2.115833	2.56E-09
1700016K19Rik	2.246011	1.66E-33	Ccrl2	2.112062	7.70E-28
4833427G06Rik	2.24287	2.03E-16	Cd14	2.041452	1.80E-14
Ccdc34	2.233768	3.49E-30	Bcl2a1b	2.006279	4.98E-13
Nme5	2.202569	3.86E-32	Cd44	1.908584	1.31E-22
1110017D15Rik	2.056407	9.52E-23	Nr4a1	1.87598	3.92E-24
